# Titin-truncating mutations associated with dilated cardiomyopathy alter length-dependent activation and its modulation via phosphorylation

**DOI:** 10.1093/cvr/cvaa316

**Published:** 2020-11-02

**Authors:** Petr G Vikhorev, Natalia N Vikhoreva, WaiChun Yeung, Amy Li, Sean Lal, Cristobal G dos Remedios, Cheavar A Blair, Maya Guglin, Kenneth S Campbell, Magdi H Yacoub, Pieter de Tombe, Steven B Marston

**Affiliations:** 1 National Heart and Lung Institute, Imperial College London, Du Cane Road, London W12 0NN, UK; 2 Heart Science Centre, Magdi Yacoub Institute, Harefield Hospital, London UB9 6JH, UK; 3 Department of Pharmacy and Biomedical Sciences, La Trobe University, Bendigo, VIC 3550, Australia; 4 School of Medical Sciences, Faculty of Medicine and Health, University of Sydney, NSW 2006, Australia; 5 Division of Molecular Cardiology and Biophysics, Victor Chang Cardiac Research Institute, Darlinghurst, NSW 2010, Australia; 6 Division of Cardiovascular Medicine, Department of Physiology, University of Kentucky, Lexington, KY, USA; 7 Department of Physiology and Biophysics, University of Illinois at Chicago, Chicago, IL, USA

**Keywords:** Dilated cardiomyopathy, Titin, Cardiac contractility and energetics, Length-dependent activation, Super- relaxed state of myosin

## Abstract

**Aims:**

Dilated cardiomyopathy (DCM) is associated with mutations in many genes encoding sarcomere proteins. Truncating mutations in the titin gene *TTN* are the most frequent. Proteomic and functional characterizations are required to elucidate the origin of the disease and the pathogenic mechanisms of *TTN-*truncating variants.

**Methods and results:**

We isolated myofibrils from DCM hearts carrying truncating *TTN* mutations and measured the Ca^2+^ sensitivity of force and its length dependence. Simultaneous measurement of force and adenosine triphosphate (ATP) consumption in skinned cardiomyocytes was also performed. Phosphorylation levels of troponin I (TnI) and myosin binding protein-C (MyBP-C) were manipulated using protein kinase A and λ phosphatase. mRNA sequencing was employed to overview gene expression profiles. We found that Ca^2+^ sensitivity of myofibrils carrying *TTN* mutations was significantly higher than in myofibrils from donor hearts. The length dependence of the Ca^2+^ sensitivity was absent in DCM myofibrils with *TTN*-truncating variants. No significant difference was found in the expression level of *TTN* mRNA between the DCM and donor groups. *TTN* exon usage and splicing were also similar. However, we identified down-regulation of genes encoding Z-disk proteins, while the atrial-specific regulatory myosin light chain gene, *MYL7,* was up-regulated in DCM patients with *TTN*-truncating variants.

**Conclusion:**

Titin**-**truncating mutations lead to decreased length-dependent activation and increased elasticity of myofibrils. Phosphorylation levels of TnI and MyBP-C seen in the left ventricles are essential for the length-dependent changes in Ca^2+^ sensitivity in healthy donors, but they are reduced in DCM patients with *TTN*-truncating variants. A decrease in expression of Z-disk proteins may explain the observed decrease in myofibril passive stiffness and length-dependent activation.

## Graphical Abstract

**Figure:**
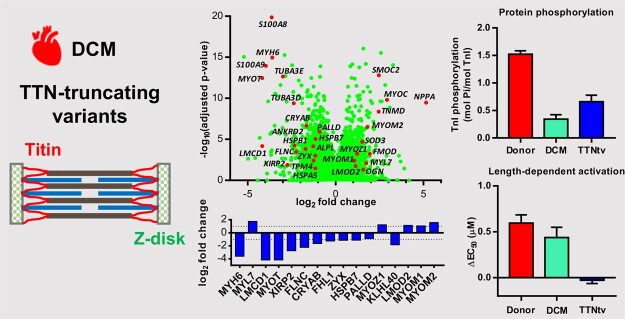

## 1. Introduction 

Heart failure is a serious, life-threatening condition.^1^ Studies have indicated that it affects ∼1.4% of the total population in the UK^2^^,^^3^ and 2.2% of adults aged 20 and older in the USA.^4^ The incidence difference between the two countries could be because of variation in the age range of the included patients and other limitations of these studies. Heart failure has two main causes^5–7^: coronary artery disease leading to infarction, and remodelling of the heart tissue, which is initially compensatory but then leads to deterioration of function and failure; and non-ischaemic heart failure (40.8% of cases in the USA^7^) with mechanisms linked to mutations in genes encoding cardiac contractile proteins. Non-ischaemic heart failure is particularly frequent in younger patients.^8^

Dilated cardiomyopathy (DCM) is a major cause of non-ischaemic heart failure.^7^^,^^9^ It is characterized by enlarged left ventricular end-diastolic dimension, decreased left ventricular ejection fraction, and reduced fractional shortening. Numerous studies have indicated that contractile dysfunction plays a central role in the initiation and progression of cardiomyopathies.^9^ DCM can also occur as a consequence of comorbidities, such as diabetes, infections, toxins (alcohol and chemotherapeutic drugs), and high blood pressure.^10^ In about half of cases, DCM is familial and is associated with more than 50 genes mostly encoding sarcomeric and cytoskeletal proteins.^11^^,^^12^

The *TTN* gene encodes the giant elastic protein titin. Truncation mutations have been discovered in up to 25% of familial DCM.^13^ Accordingly, the higher allele frequency of *TTN*-truncating variants (TTNtv) has been associated with DCM in this cohort of patients.^14^ However, the role of *TTN* mutations and how they might lead to the development of DCM is not well understood. A further complication is the observation that *TTN*-truncating variants (TTNtv) have also been detected in the healthy population (0.6–2.9%),^15^^,^^16^ where they increase the risk of development of cardiomyopathy during pregnancy,^17^ after chemotherapy,^16^^,^^18^ and in alcohol abuse.^19^ Mutations in the A-band region of titin are predominantly associated with DCM.^15^

The sarcomere is the basic contractile unit of striated muscles, including cardiomyocytes. Cardiac sarcomeres are ∼1 µm in diameter and 2.2 µm long. Myofibrils consist of repeating sarcomeres. Z-disks mark the ends of the sarcomere and are the structure that links adjacent sarcomeres together. Actin-containing thin filaments and myosin-containing thick filaments are connected to the Z-disk by α-actinin and titin. Powered by the energy of adenosine triphosphate (ATP), myosin heads interact with actin filaments to produce force. Actin filaments are associated with the troponin complex and tropomyosin that control this process in response to sarcomeric [Ca^2+^] changes. The sensitivity of myofilaments to Ca^2+^ is modulated by phosphorylation of troponin I (TnI) at Ser 23 and 24, and of myosin binding protein-C (MyBP-C) at Ser 275, 284, and 304 by protein kinase A (PKA) in response to adrenergic^20–22^ stimulation. However, Ca^2+^- and phosphorylation-dependent changes in contractility that occur during DCM are poorly understood.

The mechanical properties of titin are important for the work output of the sarcomere. Titin acts as a stretch sensor,^23^ and decreased myofibrillar stiffness has been reported in patients with DCM.^24^^,^^25^ However, the factors modulating passive myofibrillar stiffness are still under intensive investigation.^26^^,^^27^ Changes in alternative splicing of titin can alter mechanical properties and influence the binding and other properties of the translated titin molecule, and subsequently the functional properties of the heart muscle. Mutations in the gene encoding RNA binding motif protein 20 (RBM20), which regulates *TTN* splicing, has been implicated in DCM.^28^^,^^29^ Expression of a more compliant titin isoform in these patients may trigger the adverse changes leading to DCM.^30^ Additionally, phosphorylation of titin at different sites may lead to different changes in its passive stiffness.^26^^,^^31^ PKA and PKG (cGMP-dependent protein kinase) phosphorylate N2B titin domain and decrease titin stiffness.^31^^,^^32^ Whereas, PKC (protein kinase C) phosphorylates the PEVK region and increases titin stiffness.^31^ Despite extensive interest in titin and strong acknowledgement of the prevalence of TTNtv in DCM, the mechanism of the involvement of TTNtv in cardiomyopathy is still unclear. Truncated titin variants were not detected in the heart of DCM patients with TTNtv and the total titin expression was unchanged.^15^^,^^24^^,^^33^ However, cardiovascular stress may play a role in the clinical manifestation of DCM in this group of patients. Experiments with the use of a mouse model with a *TTN* truncation mutation showed that administration of angiotensin II stimulated left ventricular dilatation, systolic dysfunction, and myocardial fibrosis.^33^

The Frank–Starling law of the heart states that the force of cardiac contraction increases with diastolic volume and cardiomyocyte length.^34^ However, neither the proteins involved nor the mechanism of the length-dependent activation is currently well understood.^35^

Recently, attention has focused on the super-relaxed state of myosin,^36^ which is characterized by very slow ATPase activity. Myosin light chain kinase, via phosphorylation of the myosin regulatory light chains (MYL2), may regulate the number of myosin heads in the super-relaxed state.^36^^,^^37^ It is also possible that the super-relaxed state can be regulated via phosphorylation of MyBP-C.^38^

The objective of this research was to determine the effects of truncating mutations in the *TTN* gene on the Ca^2+^ sensitivity of force, length-dependent activation, and their modulation by PKA catalysed phosphorylation in myofibril preparations from the hearts of DCM patients (*Table 1*). We also estimated the kinetic parameters of myofibril contraction. Transcriptomic approach was used to help us understand the molecular mechanisms involved in the disease process. All titin-truncating mutations included in this study are located in the A-band region of titin.^24^

**Table 1:** Patient characteristics

Heart sample	ID	Gene	Mutation	Sex	Age (years)	Diagnosis and clinical notes
DCM						
D12	4.047	MYOM1	E247K	F	63	Familial DCM, LVEF 20%, NYHA IV, LVEDD 87 mm, LVESD 78 mm, ventricular tachycardia, dual pacemakers. No acute myocardial infarction or ischaemic heart disease, areas of full thickness fibrosis, normal coronary arteries. Severe left ventricular dilation. No diabetes.
D16	7.036			M	56	Idiopathic DCM, LVEF 5–20%. LVEDD 78 mm, LVESD 58 mm, FS 15–20%, CO 3.2 L/min, CI 1.7. Implantable cardioverter-defibrillator, ischaemic heart disease, viral, severe global dilatation. No diabetes.
DCM TTNtv						
D6	4.100	*TTN*	p.(R24390Tfs*41)	M	22	Familial DCM, six close relatives also developed DCM, post-viral cardiomyopathy, LVEF 15%, no coronary artery disease. No diabetes.
D7	4.125	*TTN*	p.(R24390Tfs*41)	M	37	Familial DCM, LVEF 15%, NYHA IV, severe dilation of all four heart chambers, severe systolic impairment of both ventricular chambers. No diabetes.
D9	2.029	*TTN*	p.(Y19850*)	F	22	Familial DCM, LVEF 13–20%, NYHA III, CO 5.1 L/min, CI 3.2 L/min/m^2^, impaired systolic, moderate LV dilation, 3-year history. Ventricular tachycardia, dual-chamber pacemaker, no myocardial infarction. Diabetes status is unknown. No ischaemia present.
D28	3.133	*TTN*	p.(N23731Kfs*5)	F	60	Familial DCM, LVEF 25%, LVEDD 64 mm, LVESD 54 mm, FS 16%, CO 1.9 L/min, CI 1.1 L/min/m^2^, diagnosis 9 years, atrial fibrillation 6 months. Diabetes status is unknown. Possible ischaemia with signs of previous infarcts and occluded epicardial artery.

*TTN* mutations are numbered according to Refseq NP_001243779.

CI, cardiac index; CO, cardiac output; FS, fractional shortening; LVEDD, left ventricular end-diastolic diameter; LVEF, left ventricular ejection fraction; LVESD, left ventricular end-systolic diameter; NYHA, New York Heart Association.

## 2. Methods

### 2.1 Patient and donor clinical characteristics

Left ventricular tissue was obtained from explanted hearts of patients diagnosed with familial or idiopathic DCM (4.100, 4.125, 2.029, 3.133, 4.047, 7.036, 2.008, 4.121) from the Sydney Heart Bank.^39^ Donor hearts had no history of cardiac disease and were obtained when no suitable transplant recipient was found (Supplementary material online, *Table S1*). All samples from heart transplant patients were cryopreserved within minutes of the loss of coronary circulation. Donor heart samples were from the University of Kentucky (24713, CF462, D0F54, D612E, BC90C, 4B3FA) and the Sydney Heart Bank (5.138, 5.089, 5.128, 5.090, 6.008, 4.083, 5.131, 4.104, 5.084, 7.080, 5.003, 5.054, 5.126, 5.048, 5.086). Patients provided written informed consent under ethical approvals obtained by the University of Sydney and the University of Kentucky. The investigation conformed with the principles outlined in the Declaration of Helsinki. Human research ethics approval was obtained from the NHS National Research Ethics Service, South West London REC3 (10/H0803/147); the Imperial College Healthcare Tissue Bank (HTA license 12275, REC approval 17/WA/0161); the University of Sydney (HREC #2012/2814); and the University of Kentucky, USA (08-03338-F2L). The mutations in the *TTN* gene were discovered earlier by whole-exome sequencing.^40^ Fifty-eight genes implicated in DCM were screened for potentially disease-causing variants, and no other mutations were found in the studied cohort of patients with TTNtv,^40^ suggesting that TTNtv are the most likely cause of DCM in these patients.

### 2.2 mRNA sequencing

mRNA extraction, quality control, library preparation, and sequencing (Illumina HiSeq 2x150 bp, to an average depth of approximately 54 million reads per sample) were performed by GENEWIZ (South Plainfield, NJ, USA). Sequencing quality and mapping statistics are summarized in the Supplementary material online, *Table S2*. DESeq2^41^^,^^42^ was used to compare gene expression between the healthy donor (*n* = 6; mean age 33 ± 10 years, 50% male) and patient groups of samples (*n* = 4; mean age was 35 ± 9 years, 50% male). The data were analysed for alternative splicing using DEXSeq.^43^ RNA-Seq reads were mapped to the human hg38 genome assembly.

### 2.3 Gel electrophoresis and western blotting

Phos-tag SDS polyacrylamide gel electrophoresis and western blotting were performed using standard methods.^22^^,^^44^ The primary antibodies used for western blotting were cardiac anti-TnI mouse monoclonal antibody P4-14G5 (ThermoFisher/Invitrogen, MA1-20119), anti-MyBPC3 mouse monoclonal antibody G-7 (Santa Cruz, sc-137237), and anti-myosin light chain 2 rabbit monoclonal antibody (Abcam, ab183490). The membranes were treated with HRP-linked secondary anti-mouse (GE Healthcare, NA931) or anti-rabbit (Abcam, ab205718) antibodies and visualised using ECL western blotting detection reagent (Amersham) or SuperSignal West Pico PLUS Chemiluminescent Substrate (Thermo Fisher Scientific). Images were recorded with the Syngene G: box gel documentation system and analysed using GeneTools software. Phosphatase inhibitor PhosSTOP (Roche) was added during sample preparation to preserve MYL2 phosphorylation.

### 2.4 Preparation of single myofibrils

Myofibrils were prepared from frozen left ventricular tissue according to a published procedure.^45^ Myofibrils were stored in rigor solution on ice until use within 2 days.

### 2.5 Phosphorylation and dephosphorylation of myofibrils in vitro

Myofibrils were phosphorylated with PKA catalytic subunits from bovine heart (Merck, P2645; 500 Units/mL) at 20°C for 20 min in a solution containing (mmol/L): 10 MOPS (pH 7.0), 10 EGTA, 5 DTT, 5 Mg-ATP, and 1 free Mg^2+^. Protein dephosphorylation was achieved using incubation with λ phosphatase (New England Biolabs, P07503; 1000 U/mL) at 20°C for 40 min in a solution containing (mmol/L): 10 MOPS (pH 7.0), 5 DTT, 3 Mn^2+^, 0.1 Mg^2+^, and 20 2,3-butanedione monoxime. Following incubation, myofibrils were pelleted, the supernatant removed, and the myofibril pellet resuspended and stored in rigor solution: 10 Tris (pH 7.1), 132 NaCl, 5 KCl, 1 MgCl_2_, 5 EGTA, 5 dithiothreitol (DTT), and 10 NaN_3_. All solutions were supplemented with protease inhibitors (μmol/L): 10 chymostatin, 5 pepstatin, 40 leupeptin, 10 E-64, and 200 PMSF.

### 2.6 Single myofibril mechanics

The apparatus for the measurement of force and passive stiffness in single myofibrils has been previously described.^45^ A single myofibril or small bundle was suspended horizontally using two specially prepared glass microtools. Contraction and relaxation were initiated by a rapid Ca^2+^ concentration jump achieved by a fast solution switch system. The mechanical force data were fit to the Hill equation: *F* = *F*_0_ + *F*_max_[Ca^2+^]^nH^/(${\text{EC}}_{50}^{\text{nH}}$ + [Ca^2+^]^nH^), where *F* is steady-state developed force, *F_max_* is the maximum saturated value F can attain, EC_50_ is the concentration of Ca^2+^ at which *F* attains 50% of *F_max_*, and *n*_H_ is the Hill coefficient. Information on myofibril kinetics is provided in Supplementary material online, *Methods*. Measurement of passive force was performed as described previously.^24^ Experiments were performed at 17°C.^46^

Relaxing (0.1 µmol/L free Ca^2+^) and activating (0.4–15.8 µmol/L free Ca^2+^) solutions contained (in mmol/L): 10 MOPS (pH 7.0), 5 Mg-ATP, 1 free Mg^2+^, 5 DTT, 10 phosphocreatine, 0.5 mg/mL creatine kinase, 1 unit/mL bacterial purine nucleoside phosphorylase, and 0.5 7-methylguanosine. The Ca-EGTA:EGTA ratio was set to obtain 10 mmol/L total EGTA and the desired free [Ca^2+^]. K-propionate and Na_2_SO_4_ were added to adjust the ionic strength of the solution to 200 mmol/L. The relaxing solution in the bath chamber (0.01 µmol/L free Ca^2+^) was supplemented with (in μmol/L): 10 chymostatin, 5 pepstatin, 40 leupeptin, 10 E-64, and 200 PMSF.

### 2.7 Simultaneous measurement of force and myosin ATPase in cardiac strips

The experimental apparatus for simultaneous measurements of force and myosin ATPase in cardiac fine strips have previously been described in detail.^47^ More information is provided in the Supplementary material online.

### 2.8 Statistical analysis

Statistics analysis and graphs were prepared using Prism 7 (GraphPad Software, San Diego, CA, USA). Data are expressed as means ± SEMs. One-way ANOVA followed by Fisher’s least significant difference multiple comparison test was used for multiple group comparison. The Student’s *t*-test was used to compare two groups of normally distributed variables; otherwise, the Mann–Whitney *U*-test was used. The linear mixed model analysis was used to compare patient and donor groups. The model was fit using the restricted maximum likelihood method. Patient and donor samples were entered to the model as random factors. Disease, treatment type, and sarcomere length were considered as fixed factors. The analysis was performed using SPSS Statistics software (IBM, version 26), and estimated means are reported. In this case, data are shown as estimated means ± SEMs. *P*-values of <0.05 were considered statistically significant. The Wald test was used to generate *P*-values and log2 fold changes in gene expression. Genes with an adjusted *P*-value <0.05 and absolute log2 fold change >1 were called differentially expressed genes.

## 3. Results

### 3.1 Gene expression

The four heterozygous *TTN* mutations (samples D6, D7, D9, and D28) result in premature termination of mRNA translation. We performed mRNA sequencing on the samples of left ventricular heart tissue used for the functional measurements to examine gene expression patterns and *TTN* alternative exon usage. A stop codon can lead to non-sense-mediated decay of the mRNA or production of truncated titin proteins. However, we did not find significant down-regulation of the *TTN* gene in the DCM samples (*n* = 4) with frameshift mutations compared to healthy donor heart samples (*n* = 6; Supplementary material online, *Table S3*). Additionally, the samples with *TTN* mutations were very similar to the exon usage of healthy donor hearts (Supplementary material online, *Figure S1* and *Table S3*). However, differences were observed in the expression of several other genes (Supplementary material online, *Table S4*) that might explain the aetiology. We found that the gene *MYL7* that encodes atrial-specific myosin regulatory light chain 2 (MLC2a) was up-regulated (3.4-fold of healthy donors) in the left ventricle of the DCM patients with TTNtv. In contrast, the expression of *MYH6* encoding alpha heavy chain subunit of cardiac myosin, which is also expressed predominantly in atrial tissue, decreased 12.4-fold.

The expression levels of many central genes encoding Z-disk structural proteins were significantly down-regulated in DCM patients with TTNtv compared to healthy donors: *FLNC* (filamin-C; 4.7-fold decrease), *MYOT* (myotilin; 18.1-fold), *PALLD* (palladin; 1.9-fold), *XIRP2* (xin actin-binding repeat containing 2; 6.7-fold), *ZYX* (zyxin; 2.2-fold), *CRYAB* (α-crystallin B chain; 3.2-fold), *MYOZ1* (myozenin-1; 2.3-fold), and *LMCD1* (LIM and cysteine-rich domains 1; 18-fold). *FHL1* (four and a half *LIM* domains protein 1) and *KLHL40* (kelch-like family member 40) were down-regulated 2.4 and 3.6-fold, respectively. The gene *LMOD2* (leiomodin-2),^48^ an actin-capping and length-regulating protein, was 2.2-fold up-regulated. The expression levels of *MYOM1 and MYOM2* (myomesin-1 and -2) were 2.1- and 3-fold up-regulated in DCM patients with TTNtv. Myomesin is located in the M-band and links titin to myosin filaments. Overexpression of myomesin was associated with sarcomere damage.^49^ The level of expression of *HSPB1* (heat shock protein beta-1), involved in mechano-transduction,^50^ decreased 3.3-fold. The following extracellular matrix protein genes were up-regulated in DCM patients: *MYOC* (myocilin; 7.5-fold), *FMOD* (fibromodulin; 3.8-fold), *OGN* (osteoglycin; 3.0-fold), and *SOD3* (extracellular superoxide dismutase [Cu-Zn]; 1.5-fold). The pro-inflammatory protein genes *S100A8* and *S100A9* (calprotectin) were down-regulated 12.5-fold and 15.6-fold, respectively. A marker of endoplasmic reticulum stress, *HSPA5* (heat shock protein family A member 5) was down-regulated 2.2-fold. The full list of the significantly impacted genes is shown in the Supplementary material online, *Table S4*.

### 3.2 Protein phosphorylation

**Figure 1:**
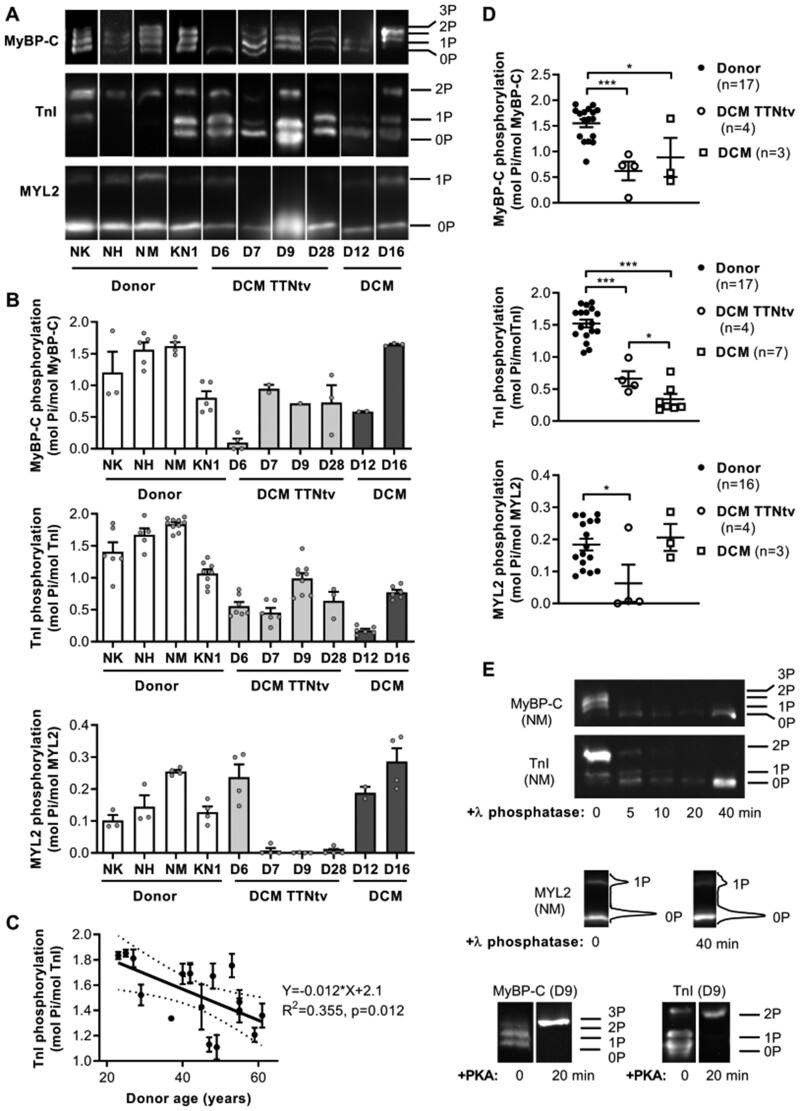
Phosphorylation level of TnI, MyBP-C and MYL2 in the left ventricular myocardium of healthy donor and DCM hearts. (*A*) Representative western blot image of donor and DCM samples with or without TTNtv. Differently phosphorylated species of TnI, MyBP-C, and MYL2 were separated by Phos-tag SDS page gel followed by Western blotting with anti-TnI, anti-MyBP-C, and anti-MYL2 antibodies. Proteins were separated according to their phosphorylation level: tris-phosphorylated (3P), bis-phosphorylated (2P), monophosphorylated (1P), and unphosphorylated form (0P). Samples are individually plotted to illustrate the range of phosphorylation across donor and DCM heart samples. (*B*) The densitometric analysis of western blots is shown below each representative western blot. (*C*) Linear regression analysis of TnI phosphorylation in donors. The scatter plot suggests that TnI phosphorylation declines with donor age. The solid line is a least-squares linear regression line with 95% confidence interval (dotted line). (*D*) The level of phosphorylation of TnI, MyBP-C, and MYL2 was significantly reduced in DCM patient samples with TTNtv. Phosphorylation levels of TnI in DCM samples 4.032, 4.081, and 3.107 (referred as FA, FC, and FD) have been reported earlier.^57^ Statistical analysis was performed using one-way ANOVA with Fisher’s least significant difference test. *P < 0.05 and ***P < 0.001. (*E*) Treatment with PKA and λ phosphatase, respectively, increased and decreased the level of phosphorylation of contractile proteins TnI and MyBP-C in myofibrils. λ phosphatase treatment decreased the MYL2 phosphorylation level in NM sample from 25.4% to 18.3%.

We performed Phos-tag gel electrophoresis, in which proteins are separated according to their phosphorylation level, and western blotting to determine the level of TnI, MyBP-C, and MYL2 phosphorylation in the samples (*Figure 1A and B*). Western blots with anti-TnI antibodies showed three bands corresponding to bis-phosphorylated (at Ser 23 and 24), monophosphorylated and unphosphorylated protein (*Figure 1A*). MyBP-C contains three accessible phosphorylated sites (Ser 275, 284, and 304) per molecule.^22^^,^^51^ Three or four different migration bands were resolved on western blots with MyBP-C antibodies (*Figure 1A*). The intensity of the fourth band (3P) was weak and seen only in the highly phosphorylated control sample NM. All other studied donor heart samples showed only three bands (0P, 1P, and 2P). The level of phosphorylation of TnI, MyBP-C, and MYL2 was significantly decreased in DCM patients with TTNtv compared to healthy donors (*Figure*  1A, B, and *D*). The level of phosphorylation of TnI but not MYL2 was also reduced in DCM samples without TTNtv (D12, 0.18 ± 0.02 mol Pi/mol TnI; D16, 0.74 ± 0.04 mol Pi/mol TnI; *Figure 1B and D*).

We observed a significant negative correlation between TnI phosphorylation and donor age (*Figure 1C*). This suggests that the TnI phosphorylation level in non-diseased hearts may naturally decline with age. DCM samples with mutations in the *TTN* gene had significantly lower levels of phosphorylation of TnI: 0.66 ± 0.12 vs. 1.52 ± 0.06 mol Pi/mol TnI (*P* < 0.001; four patient and 17 donor hearts, respectively; *Figure 1D*). The level of phosphorylation of MyBP-C and MYL2 in DCM samples with TTNtv was 0.62 ± 0.18 vs. 1.55 ± 0.08 mol Pi/mol MyBP-C (*P* < 0.001; four patient and 17 donor hearts, respectively; *Figure 1D*) and 0.06 ± 0.11 vs. 0.18 ± 0.02 mol Pi/mol MYL2 (*P* < 0.05; four patient and 16 donor hearts, respectively; *Figure 1D*).

In the diseased myocardium, the β adrenoceptor is often down-regulated via receptor phosphorylation and β-arrestin binding.^52^ This blunts the response to β-adrenoceptor activation via sympathetic stimulation and thus PKA-induced phosphorylation of contractile proteins. To distinguish the effect of mutation from possible effects caused by dephosphorylation of TnI and also to understand how phosphorylation of TnI is associated with heart disease and may change its progression, we manipulated its phosphorylation level in our samples. The PKA catalytic subunit was used to increase the TnI phosphorylation level in DCM myofibrils. In contrast, λ phosphatase may be used to dephosphorylate TnI and MyBP-C.^53^^,^^54^ Phosphorylation with PKA resulted in fully phosphorylated TnI (2P per molecule) and MyBP-C (3P per molecule; *Figure 1E*). Treatment with λ phosphatase (1000 U/mL, 40 min at 20°C) resulted in fully dephosphorylated TnI and MyBP-C (*Figure 1E*), and a decrease (∼27%) in MYL2 phosphorylation (*Figure 1E*).

### 3.3 Myofilament Ca^2+^ sensitivity and TnI phosphorylation

Myofibril contractility can be described by several parameters: the maximum force of isometric contraction, the Ca^2+^ sensitivity of force production, the rates of force development and relaxation and their modulation by sarcomere length, and TnI and MyBP-C phosphorylation levels. The maximum force, length dependence of maximal force, kinetics of muscle contraction and relaxation, and passive stiffness have been studied previously in DCM heart samples with truncating mutations.^24^ Our study focused on the Ca^2+^ sensitivity of force development, its length dependence and the possible functional role of changes in phosphorylation of contractile proteins in the disease. We also verified that myofibrils isolated from an additional DCM sample with TTNtv, D28, had decreased passive stiffness. The Young’s modulus was 45.6% lower in D28 compared to donor heart myofibrils, similar to the values found with the other TTNtv DCM samples^24^ (Supplementary material online, *Figure S2*).

Four DCM samples with TTNtv were compared with left ventricular samples of four donor hearts: NM, young adult (23 years old); KN1, middle-aged adult (47 years old), NH (48 years old), and KN2 (61 years old). TnI and MyBP-C were highly phosphorylated in donor NM (1.80 ± 0.05 mol Pi/mol TnI and 1.62 ± 0.06 mol Pi/mol MyBP-C) and NH (1.6 ± 0.1 mol Pi/mol TnI and 1.56 ± 0.11 mol Pi/mol MyBP-C), but not in KN1 (1.18 ± 0.04 mol Pi/mol TnI and 0.80 ± 0.11 mol Pi/mol MyBP-C). These differentially phosphorylated healthy donor samples were used to distinguish the effect of dephosphorylation of TnI and MyBP-C regulatory proteins in DCM samples from other effects caused by mutations. The relatively low phosphorylated control sample KN1 was used in functional measurements for the donor–patient comparison. Furthermore, we treated isolated myofibrils with λ phosphatase and PKA to change phosphorylation levels of TnI and MyBP-C.

**Figure 2:**
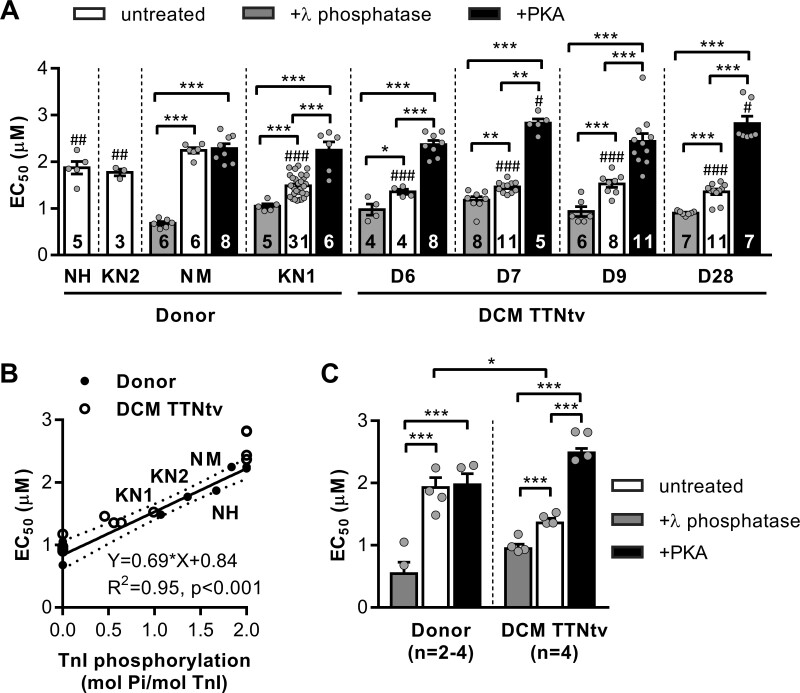
Ca^2+^ sensitivity of force in cardiac myofibrils and its modulation via TnI phosphorylation. (*A*) The graph shows the concentrations of Ca^2+^ required for half-maximal force for donor and DCM patient hearts with truncating mutations in the *TTN* gene. Cardiac myofibrils were treated with PKA and λ phosphatase to fully phosphorylate and dephosphorylate TnI, respectively. Statistical analysis was performed using one-way ANOVA with Fisher’s least significant difference test. ^#^*P* < 0.05, ^##^*P* < 0.01, and ^###^*P* < 0.001 vs. donor NM. *P < 0.05, **P < 0.01, and ***P < 0.001 vs. no treatment or other treatment. Numbers on bars indicate number of myofibril samples. (*B*) Correlation between myofilament Ca^2+^ sensitivity and TnI phosphorylation. The EC_50_ for Ca^2+^ required for half-maximal force responses in untreated, PKA-, and λ phosphatase-treated cardiac donor myofibrils (closed dots) are plotted against TnI phosphorylation level. The solid line is a least-squares linear regression line with 95% confidence interval (dotted line). Pearson correlation coefficient *r* = 0.91, *n* = 8 donor hearts (four untreated, two PKA-treated and two λ phosphatase-treated). The open circles are for DCM myofibrils (untreated, PKA-, and λ phosphatase-treated). (*C*) The EC_50_ values for the combined group of DCMs with TTNtv vs. healthy donors. Sarcomere length was 2.2 µm. Statistical analysis was performed using linear mixed model. Bars show estimated marginal means ± SE. Grey circles represent mean values of individual heart samples. *P < 0.05 and ***P < 0.001. Measurements were performed at 17°C.

The maximum tension response to different [Ca^2+^] was fit with the Hill equation to calculate the EC_50_, [Ca^2+^] required to reach half-maximal force response. The EC_50_ for donor heart myofibrils was consistent with the TnI phosphorylation level. Myofibrils with a lower TnI phosphorylation level had a higher Ca^2+^ sensitivity (lower EC_50_). The EC_50_ values for donor heart samples NM, NH, KN1, and KN2 were 2.24 ± 0.06, 1.87 ± 1.14, 1.48 ± 0.04, and 1.77 ± 0.07 μmol/L, respectively (*Figure 2A*). The Ca^2+^ sensitivities of DCM samples with truncating mutations in the *TTN* gene were significantly higher than those of donor hearts NM (*Figure 2A*) and NH (*P* < 0.038), but with no significant difference compared to KN1 (*Figure 2A*). The EC_50_ values for DCM with TTNtv were 1.36 ± 0.05 μmol/L for D6, 1.46 ± 0.04 μmol/L for D7, 1.53 ± 0.08 μmol/L for D9, and 1.36 ± 0.06 μmol/L for D28.

The changes in Ca^2+^ sensitivity were coupled to the changes in TnI phosphorylation level (*Figure 2B*). Treatment with PKA (fully phosphorylated TnI) significantly decreased myofilament Ca^2+^ sensitivity for donor KN1 and DCM with TTNtv samples, and treatment with λ phosphatase (fully unphosphorylated Tn-I) significantly increased myofilament Ca^2+^ sensitivity for all samples (*Figure 2A and C*). The difference between EC_50_ values was not significant between samples of either treatment group when determined at the same phosphorylation level (*Figure 2C*). However, the EC_50_ value for untreated DCM myofibrils with TTNtv was significantly lower compared to healthy donor heart myofibrils (1.36 ± 0.08 vs. 1.93 ± 0.16 μmol/L, *P* < 0.05; four patient and four donor hearts; *Figure 2C*, white bars).

The maximal active tension depended on the sarcomere length, and is a function of overlap between myosin and actin filaments. Treatment with PKA and λ phosphatase did not affect significantly (*P* > 0.05, for all sarcomere lengths) the maximum force of isometric contraction (Supplementary material online, *Figure S3*) compared to corresponding untreated samples.

The decline in TnI phosphorylation in DCM hearts is adaptive in the short term as it helps to increase the force of heart contraction. However, such functional tuning can become maladaptive in the long term as it reduces the inotropic reserve of the heart in response to β-adrenergic stimulation.^55^

### 3.4 Length-dependent activation

**Figure 3:**
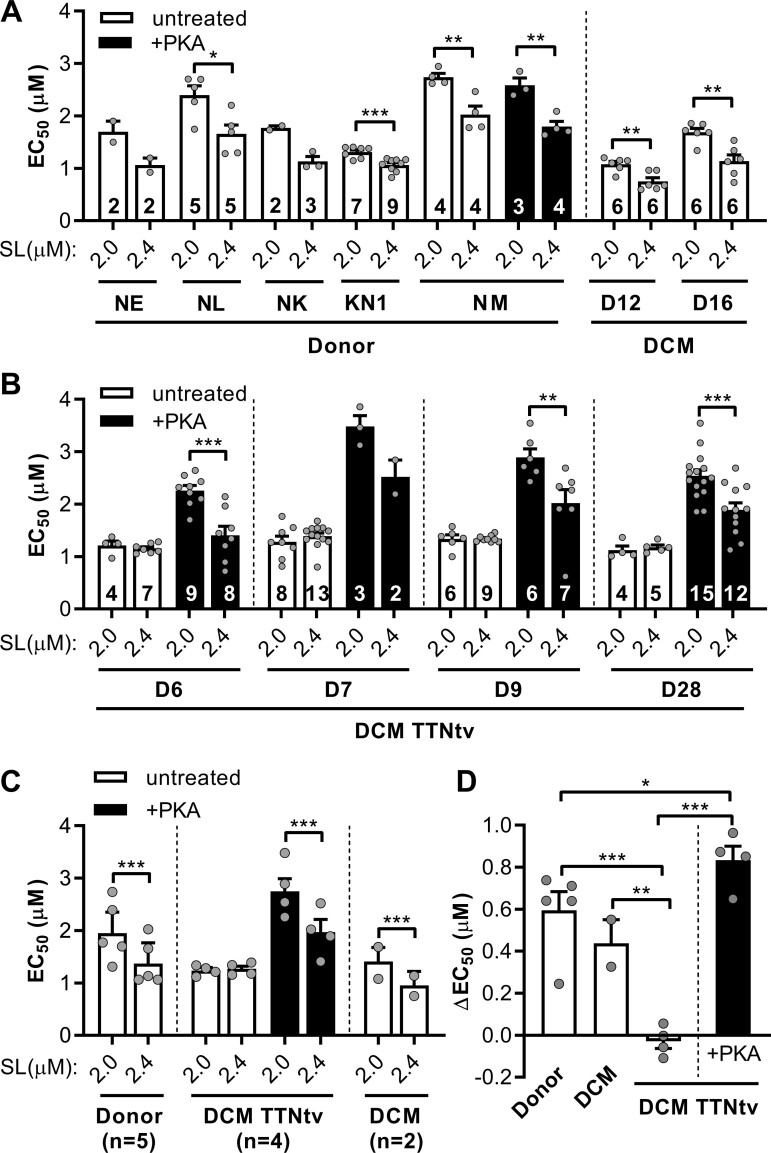
Length dependence of the Ca^2+^ sensitivity of force. The maximum force was measured at different concentrations of Ca^2+^ and the EC_50_ required for the half-maximal activation was calculated. The measurements were performed at short (2.0 µm) and long (2.4 µm) sarcomere lengths (SL). (*A*) The calcium sensitivity of force (EC_50_) in donor heart myofibrils and DCM myofibrils without TTNtv. A significant shift in EC_50_ was observed both in low- and high-phosphorylated healthy donor heart and DCM without TTNtv samples. (*B*) Mutations in titin abolished the length-dependent changes in EC_50_. The changes in EC_50_ by stretch were restored by PKA-induced phosphorylation. Statistical analysis was performed using Student’s *t*-test or the Mann–Whitney *U* test. *P < 0.05, **P < 0.01, and ***P < 0.001. Numbers on bars indicate number of myofibril samples. (*C*) Statistics for the combined group of DCMs and healthy donors. Statistical analysis was performed using linear mixed model. Bars show estimated marginal means ± SE. Grey circles represent mean values of individual heart samples. (*D*) The difference between the EC_50_ values measured at 2.0 µm and 2.4 µm. Statistical analysis was performed using one-way ANOVA with Fisher’s least significant difference test. Grey circles represent mean ΔEC_50_ values of individual heart samples. Measurements were performed at 17°C.

Stretching of cardiac myofibril from 2.0 to 2.4 µm significantly decreased the concentration of Ca^2+^ required for half-maximal force production. Control samples showed a shift in force vs. [Ca^2+^] curve after changing sarcomere length (*Figure 3A*, untreated). The shift was greater in the control sample NM (ΔEC_50_ = 0.712 ± 0.179 μmol/L) with more highly phosphorylated TnI than in the sample KN1 (ΔEC_50_ = 0.245 ± 0.058 μmol/L; *Figure 3A*). The mean value of ΔEC_50_ for healthy donor heart myofibrils was 0.583 ± 0.164 μmol/L (*n* = 5 hearts, linear mixed model analysis; *Figure 3C*). In contrast, length dependence of Ca^2+^ sensitivity was not seen in four DCM samples with TTNtv (ΔEC_50_ = −0.037 ± 0.051 μmol/L, *n* = 4 hearts; *Figure 3C*). Importantly, we showed that the length dependence of EC_50_ was preserved in control sample KN1 (*Figure 3A*, white bars), with a low phosphorylation level of both TnI (1.18 ± 0.04 mol Pi/mol TnI) and MyBP-C (0.80 ± 0.11 mol Pi/mol MyBP-C). Moreover, the shift in EC_50_ was also observed in DCM samples D12 and D16 with a lower level of TnI phosphorylation but without TTNtv (ΔEC_50_ = 0.403 ± 0.089 μmol/L, *n* = 2 hearts; *Figure 3C*).

To determine whether the decrease in phosphorylation can affect length-dependent activation, we used samples with different levels of phosphorylation of TnI: native and fully phosphorylated by PKA. Decreased level of TnI phosphorylation in heart failure samples correlates with a decreased length-dependent activation.^56^ PKA treatment restored length-dependent changes in EC_50_ in DCM with TTNtv (ΔEC_50_ = 0.78 ± 0.12 μmol/L, *n* = 4 hearts; *Figure 3C*, black bars). This value is similar to the values we found for the highly phosphorylated control NM (ΔEC_50_ = 0.71 ± 0.18 μmol/L for untreated NM and ΔEC_50_ = 0.79 ± 0.16 μmol/L for PKA-treated NM; *Figure 3A*). However, in the case of DCM with TTNtv, length-dependent activation was not present in sample D9, with phosphorylation levels of TnI and MyBP-C similar to control KN1, as well as in other samples with TTNtv (*Figure 3B*, white bars). Also, length-dependent activation was present in samples D12 and D16 (*Figure 3A*), where the phosphorylation level of TnI was lowest.

We concluded that a certain level of phosphorylation of TnI is essential, but not enough for normal length-dependent activation. *TTN* mutations led to decreased length-dependent activation independently of the dephosphorylation of TnI and MyBP-C. Dephosphorylation of regulatory proteins during disease progression and ageing can only cause further impairment of both length-dependent activation and the rate of contraction.

## 4. Discussion

Phosphorylation levels of TnI and MyBP-C are usually reduced in cardiomyopathies.^22^^,^^56–59^ The reason for this is that diseased myocardium β adrenoceptors are often down-regulated via receptor phosphorylation and β-arrestin binding.^52^ Phosphorylation levels of TnI and MyBP-C are decreased in idiopathic DCM^58^^,^^59^ and normal in a number of human DCM samples with mutations in thin-filament protein genes (*TNNI3, TNNT2, TNNC1*),^59^^,^^60^ but reduced in DCM samples with TTNtv (*Figure 1D*). Moreover, phosphorylation of MYL2 was significantly reduced in DCM with TTNtv (*Figure 1D*); three of four studied samples were virtually unphosphorylated (*Figure 1B*). This is in contrast to idiopathic DCM, not associated with TTNtv, which showed MYL2 phosphorylation level comparable to donor hearts.^61^^,^^62^

Higher Ca^2+^ sensitivity in DCM muscles with mutations in *TTN* (*Figure 2C*) was associated with a reduced phosphorylation level of TnI compared to donor hearts (*Figure 1D*). The Ca^2+^ sensitivity in DCM myofibrils was modulated via TnI phosphorylation (*Figure 2B*). Myofibrils with higher levels of phosphorylation had faster cross-bridge kinetics, higher rates of contraction (*k_ACT_*; Supplementary material online, *Figure S5B*), and relaxation (lower *t_LIN_* and higher *k_REL_*; Supplementary material online, *Figure S5C* and *E*). Interestingly, our data suggest that TnI phosphorylation level may naturally decline with age (*Figure 1C*).

Length-dependent activation is the basis for the Frank–Starling law, which states that the force of contraction increases with diastolic volume and therefore with the length of the cardiomyocytes.^34^ However, the mechanism of length-dependent activation is still not well understood.^35^ The length-dependent shift in calcium sensitivity was absent in DCM myofibrils with *TTN*-truncating mutations (*Figure 3B and C*, white bars).

DCM mutations in *TTN* lead to a totally blunted length-dependent shift in the Ca^2+^ sensitivity of contraction and therefore impaired Frank–Starling mechanism. This provides a distinct mechanism of TTNtv, compared to DCM caused by other factors. In our study, we clearly revealed no difference in ΔEC_50_ (*Figure 3D*), or ratio of EC_50_ (Supplementary material online, *Figure S6*) values measured at short and long sarcomere lengths in DCM samples with TTNtv, compared to donor hearts. We would like to point out that the ratio of EC_50_ values measured at short and long sarcomere lengths is a more appropriate parameter to evaluate changes in calcium sensitivity. The EC_50_ mean ratio for DCM with TTNtv was 0.98 ± 0.03 (*n* = 4 hearts; Supplementary material online, *Figure S6*); otherwise the ratio for donor hearts was much higher, at 1.44 ± 0.07 (*n* = 5 hearts; Supplementary material online, *Figure S6*). In other studies, length-dependent activation (ΔEC_50_) has been found unchanged in ischaemic cardiomyopathy and not significantly decreased in other DCM samples, and the EC_50_ ratio between the donor and DCM patient cardiomyocytes not different.^59^^,^^63^^,^^64^ PKA effectively restored length-dependent changes in myofilament Ca^2+^ sensitivity in DCM hearts with TTNtv (*Figure 3D*).

We cannot exclude that phosphorylation of TnI modulates the length-dependent changes in Ca^2+^ sensitivity.^65^ However, the low level of TnI phosphorylation in DCM TTNtv myofibrils still does not fully explain the loss of response to stretch. Indeed, the stretch-activated Ca^2+^ sensitization was preserved in the donor heart sample KN1, with the low level of phosphorylation of both TnI and MyBP-C, and in DCM samples D12 and D16 (*Figure 3A*). We propose that a certain level of TnI phosphorylation is required for normal stretch-activated Ca^2+^ sensitization, but this phosphorylation threshold is increased in DCM muscle with TTNtv. Therefore, a reduced TnI phosphorylation in TTNtv patients is not sufficient for normal length-dependent activation. PKA phosphorylation may thus compensate for the loss of function. The decrease in phosphorylation occurring during ageing and disease progression worsens heart functionality and can lead further to heart failure.

The possible factors that modulate the passive stiffness of myofibrils (selective phosphorylation of titin and its alternative splicing) are still under investigation.^26^ PKA phosphorylates titin in the N2B domain (Ser 4065 and 4185) and decreases passive stiffness.^31^ Interestingly, decreased passive stiffness was associated with decreased length-dependent activation.^66^ It may indirectly suggest that phosphorylation of titin by PKA is not the factor increasing length-dependent activation. However, there are not enough studies on the role of titin phosphorylation in length-dependent activation. Previously, we showed that DCM with TTNtv and donor samples have similar titin protein phosphorylation levels, long to short titin isoform ratios (N2BA/N2B) and no expression of truncated titin protein variants,^24^ which is consistent with earlier studies.^15^^,^^33^ N2BA/N2B ratios are found unchanged in familial DCM^24^ but increased in patients with idiopathic DCM.^24^^,^^25^^,^^64^^,^^67^ Mutation in the *RBM20* gene caused decreased passive stiffness of titin as well as length-dependent activation.^64^

We consider a complex interplay between titin passive stiffness, stretch-induced structural changes in myosin, troponin, myosin-binding protein C,^68^ and the phosphorylation status of TnI and MyBP-C on the length-dependent changes in Ca^2+^-sensitivity.

To understand further the mechanism of influence of titin mutations on length-dependence activation, we performed mRNA sequencing of four DCM samples with truncation mutations in the *TTN* gene. We did not find a significantly different expression of *TTN* mRNA compared to healthy donors. *TTN* exon usage and splicing were also very similar (Supplementary material online, *Figure S1* and *Table S3*).

Nevertheless, mutations in the *TTN* gene may have a significant impact on the earlier stages of heart development, e.g. pre- and postnatal, and lead to disruption of the expression of proteins interacting with titin in the adult heart. Also, cardiovascular stress may play a role in the clinical manifestation of DCM in this group of patients. Increased intracellular Ca^2+^ concentration, cellular, and endoplasmic reticulum stress inhibit nonsense-mediated mRNA decay.^69^ This increases expression of truncated proteins and, in our case, stress may induce temporary expression of truncated titin variants at early stages of the disease.

We found a strong down-regulation of mRNA expression of numerous cytoskeletal proteins associated with the Z-disk. This may not be a coincidence and indeed may have important functional consequences. Many of these genes encoding Z-disk proteins are associated with the so-called myofibrillar myopathies^70–72^ and DCM.^12^ The Z-disk is important for mechanical stability, mechanotransduction, and signalling.^73^ Both actin and titin filaments are embedded in the Z-disk of sarcomeres via TCAP, α-actinin, and other proteins.^23^^,^^74^^,^^75^ It implies that Z-disk proteins might modulate myofibril contractility. The decreased expression of Z-disk proteins may lead to a disruption of the Z-disk structure. Therefore, we believe that loss of Z-disk integrity may explain the observed decrease in myofibril passive stiffness and length-dependent activation. Indeed, we found that the length-dependent changes in Ca^2+^ sensitivity observed in healthy donor heart samples were absent in heart samples with mutations in the *TTN*. Thus, titin contributes to the Frank–Starling mechanism not only as a passive elastic component but also as an active regulator of actin–myosin interaction (*Figure 5*).

**Figure 4:**
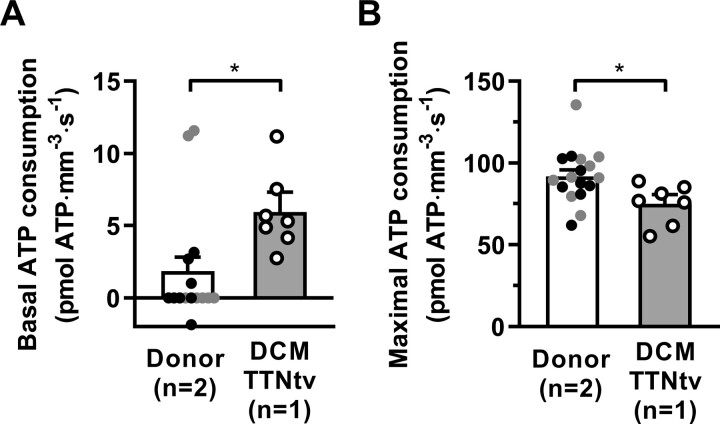
ATP consumption rate in skinned cardiac strips. Simultaneous measurement of force production and ATP consumption rate by myosin measured in skinned cardiac strips. (*A*) Basal ATP activity measured in a relaxing solution containing 10 mM EGTA and no calcium. (*B*) ATP consumption achieved at maximal Ca^2+^ stimulation. The results gained for DCM heart sample D28 (6–7 muscle strips) were compared to two healthy donor hearts, NM and KN1 (black and grey circles, respectively; 15–17 muscle strips). Statistical analysis was performed using linear mixed model. Bars show estimated marginal means ± SE. Grey circles represent mean values of individual heart samples. *P < 0.05 and ***P < 0.001. Sarcomere length was 2.2 µm. Measurements were performed at 25°C.

We measured ATPase activity in thin muscle strips under relaxing conditions (basal ATPase activity) and then at different concentrations of Ca^2+^ during isometric contraction. We cannot draw a general conclusion for this study because of the small number of samples used, but these results should stimulate further investigation in this area. Mutations in *TTN* did not change the tension cost of force generation (Supplementary material online, *Figure S7D*), but significantly decreased the maximal ATP consumption rate (*Figure 4B*) and increased basal ATPase activity of myosin (*Figure 4A*). Similarly, DCM-associated mutations in *MYH7* also decrease myosin actin-activated ATPase activity.^76^ In contrast, a hypertrophic cardiomyopathy mutation in cardiac troponin T, K280N, increases the energy cost of tension generation but does not affect resting ATP activity.^77^ Basal myosin ATPase can also be significantly increased, as shown in mouse models of HCM.^78^ Basal ATPase activity has been found similar in ventricular and atrial muscle strips.^79^ Therefore, it is unlikely that the increased expression of the atrial isoform of myosin regulatory light chain 2 is associated with the increased basal ATP activity of myosin in DCM samples with TTNtv. Increased basal myosin ATPase could be related to destabilization of the super-relaxed state. The ratio of systolic to diastolic duration in the adult human heart is ∼0.6 and 0.9 at rest and during exercise, respectively.^80^ The ratio of basal to maximal ATP consumption in DCM cardiac strips (*Figure 4A and B*) was 0.08. Therefore, we can assume that at rest, ATP use by myosin during diastole in the myocardium of DCM patients with TTNtv can be more than 12% of total ATP consumption. This is 3.6 times higher compared to the healthy myocardium (∼3%).

**Figure 5:**
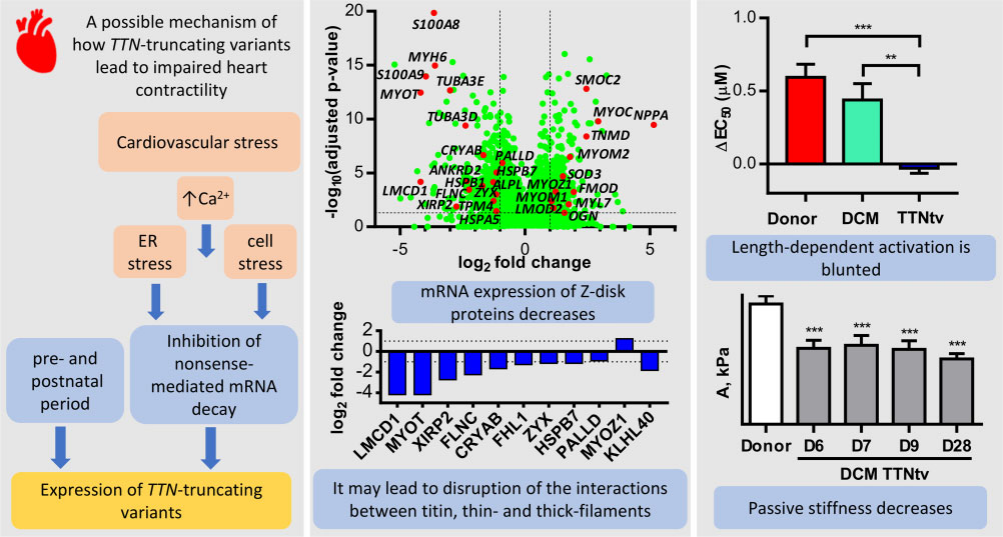
Proposed mechanism linking titin-truncating mutations with contractile dysfunction. ER, endoplasmic reticulum.

In conclusion, the results demonstrate that decreased length-dependent activation induced by mutations in the structural protein titin could be one of the most important factors in DCM pathogenesis. The increase in Ca^2+^ sensitivity, due to TnI phosphorylation level decreases, is a secondary factor that is initially adaptive but then becomes maladaptive. The decrease in length-dependent activation and reduced passive stiffness impair the Frank–Starling mechanism. Moreover, we can propose that DCM-truncating mutations in *TTN* may increase consumption of ATP during diastole that may lead to ATP depletion observed in DCM myocardium (35%)^81^ and cardiomyocyte wasting. However, the limited number of studied DCM samples with TTNtv will require follow-up work to support our conclusions in a larger cohort of patients with TTNtv.

## Supplementary material

Supplementary material is available at *Cardiovascular Research* online.

## Author contributions

P.G.V., N.N.V., and P.d.T. conceived the study or contributed to the experimental design. P.G.V., N.N.V., and W.C. performed experiments and data analysis. A.L., S.L., C.G.d.R., C.A.B., M.G., K.S.C., and S.B.M. provided human heart samples. P.G.V. and N.N.V. wrote the manuscript with help from C.G.dR. All authors discussed the results and commented on the manuscript. All authors approved the final version of the manuscript.

**Conflict of interest:** none declared.

### Funding 

The work was supported by a grant from the British Heart Foundation [PG/17/5/32705 to P.G.V. and S.B.M.]; the National Institutes of Health grant [HL-PO1 HL62426 to P.d.T.]; the Fondation Leducq [17CVD04 to M.H.Y.]; and by the Magdi Yacoub Institute [Charity number 1082750].

### Data availability

The data underlying this article are available in the article and in its online supplementary material. The raw mRNA sequencing data cannot be made publicly available due to ethical restrictions.

## Supplementary Material

cvaa316_Supplementary_Data

## Notes

Translational perspective Our findings may have implications in the development of new strategies for DCM treatment in patients with *TTN*-truncating variants as well as in the development of new drugs.
