# Supplementary material for: Titin-truncating mutations associated with dilated cardiomyopathy alter length-dependent activation and its modulation via phosphorylation
Source: Cardiovasc Res. 2020 Nov 2;118(1):241–53. doi: 10.1093/cvr/cvaa316 (PMC8752363; doi:10.1093/cvr/cvaa316)
Supplement: cvaa316_Supplementary_Data [file cvaa316_supplementary_data.zip › Supplement CVR-2019-1162R3.docx]

**SUPPLEMENTAL MATERIAL**

**Titin-truncating mutations associated with dilated cardiomyopathy alter length-dependent activation and its modulation via phosphorylation**

Petr G. Vikhorev^1^, Natalia N. Vikhoreva^2^, WaiChun Yeung^1^, Amy Li^3,4^, Sean Lal^3^, Cristobal G. dos Remedios^3^, Cheavar A. Blair^5^, Maya Guglin^5^, Kenneth S. Campbell^5^, Magdi H. Yacoub^1^, Pieter de Tombe^1,2,6^, Steven B. Marston^1^.

^1^ National Heart and Lung Institute, Imperial College London, London, W12 0NN, UK;

^2^ Heart Science Centre, Magdi Yacoub Institute, Harefield Hospital, London UB9 6JH, UK;

^3^ Discipline of Anatomy and Histology, Bosch Institute, University of Sydney, Sydney, NSW 2006, Australia;

^4^ Department of Pharmacy and Biomedical Sciences, La Trobe University, Bendigo, VIC 3550, Australia;

^5^ Division of Cardiovascular Medicine, Department of Physiology, University of Kentucky, Lexington, Kentucky, USA;

^6^ Department of Physiology and Biophysics, University of Illinois at Chicago, Chicago, Illinois, USA;

**Supplemental Methods**

**Supplemental Results**

**Supplemental Figures**

**Figure S1.** Differential exon usage in the *TTN* gene.

**Figure S2.** Myofibril passive stiffness.

**Figure S3.** Myofibril maximal force.

**Figure S4.** Kinetic parameters of myofibril contraction and relaxation.

**Figure S5.** Summary of kinetic parameters of myofibril contraction and relaxation.

**Figure S6.** Length dependence of the Ca^2+^ sensitivity of force.

**Figure S7.** Force and calcium sensitivity in skinned cardiac strips.

**Supplemental Tables**

**Table S1.** Donor characteristics.

**Table S2.** Sample sequencing statistics.

**Table S3.** Differential *TTN* exon usage results.

**Table S4** is supplied as an Excel datasheet. Significant differential gene expression analysis. Expression is shown in transcripts per million (TPM). Healthy donors (n = 6); DCM patients with TTNtv (n = 4): D6, D7, D9 and D28.

**Supplemental References**

**Supplemental Methods**

***Single myofibril mechanics (kinetics)***

The rate of force increase, the maximum achieved isometric force, and the slow and fast phase component rates of relaxation were measured^1, 2^. The rate constants for the exponential force development (*k_ACT_*) and for the fast exponential relaxation (*k_REL_*) were evaluated by curve-fitting using the equation y(t) = y_plateau_ + (y_0_ - y_plateau_) · exp (- kt). The slow relaxation phase of duration *t_LIN_* was characterised by the rate constant *k_LIN_*, calculated from the slope value of the linear regression fit by normalising it to the maximum tension.

***Simultaneous measurement of force and myosin ATPase in cardiac strips***

To prepare muscle fibres, approximately 10-mg pieces of frozen tissues were skinned overnight with 1% Triton X-100 in a relaxing solution (mmol/L): 10 EGTA, 7.45 K-propionate, 5.76 Na_2_ATP, 6.93 MgCl_2_, 100 BES, 5 NaN_3_, 1 DTT, 10 PEP, 0.03 leupeptin, 0.01 oligomycin, 0.1 PMSF, 0.01 A_2_P_5_ (Sigma-Aldrich, D4022) and protease inhibitor mixture, pH 7.0. Fine cardiac strips (diameter ~0.2 mm) were dissected from skinned heart tissue samples.

Muscle bundles were attached to a force transducer and a motor arm in the experimental apparatus via aluminium T-clips, as previously described^3^. Sarcomere length was measured by laser diffraction in the relaxing solution and set to 2.2 µm. The ATP consumption rate was normalised to muscle volume and plotted versus the steady-state force per cross-sectional area attained during each contraction and fit by linear regression; a slope of this relationship reflects tension cost, a parameter proportional to the average cross-bridge detachment rate^4^. Measurements were performed at 25 ± 0.2 °C^5^.

The relaxing solution contained (mmol/L): 10 EGTA, 7.45 K-propionate, 5.76 Na_2_ATP, 6.93 MgCl_2_, 100 BES, 1 DTT, 10 PEP, pH 7.0 and 0.01 A_2_P_5_. The activating solution contained (mmol/L): 10 Ca-EGTA, 7.45 K-propionate, 5.83 Na_2_ATP, 6.61 MgCl_2_, 100 BES pH 7.0, 1 DTT, 10 PEP and 0.01 A_2_P_5_. Each activation was preceded by a two-minute incubation in pre-activating solution (mmol/L): 0.5 EGTA, 19.5 hexamethylenediamine-N, N, N, N-tetraacetate (HDTA), 7.45 K-propionate, 5.76 Na_2_ATP, 6.67 MgCl_2_, 100 BES, 1 DTT, 10 PEP and 0.01 A_2_P_5_. Solutions with varying concentrations of free [Ca^2+^] were prepared by mixing the relaxing and activating solutions as previously described^4^. Basal ATP consumption was measured in the relaxing solution for approximately four minutes to eliminate spontaneous fluctuations and measurement noise. All solutions contained (in mmol/L) 0.5 mg/mL pyruvate kinase (386 U/mg) and 0.05 mg/mL lactate dehydrogenase (880 U/mg; Sigma-Aldrich, St. Louis, MO), 1 NADH (Roche), 5 NaN_3_, 0.03 leupeptin, 0.01 oligomycin, 0.1 PMSF, and protease inhibitor cocktail (Sigma-Aldrich, P8340).

**Supplemental Results**

***Kinetics of contraction and TnI phosphorylation***

The fast kinetics of force development (*k*_ACT_) and relaxation (*k*_LIN_, *t*_LIN_ and *k*_REL_) were measured (Supplementary material online, Figure S5A) after a switch of relaxing solution to activating solution at a submaximal concentration of Ca^2+^ (5.01–15.8µmol/L free Ca^2+^). KN1, with lower phosphorylation levels of TnI and MyBP-C, had significantly lower *k_ACT_* and longer *t*_LIN_, compared to the control NM with highly phosphorylated TnI and MyBP-C (Supplementary material online, Figures S4A and S4C). The kinetic parameters *k*_ACT_*, t*_LIN_ and *k*_REL_ were not significantly different between donor (n = 2 hearts) and DCM with TTNtv (n = 4 hearts) myofibrils (Supplementary material online, Figures S5B– 3D). Activation and relaxation kinetics in DCM with TTNtv myofibrils regardless of mutation were not significantly altered compared to control KN1 with similar phosphorylation of TnI and MyBP-C^1, 6^ (Supplementary material online, Figures S4A– S4D). However, *k*_ACT_ significantly decreased when compared to highly phosphorylated sample NM (Supplementary material online, Figure S4A). We also observed that the duration of the slow relaxation phase was substantially longer in DCM with TTNtv compared to NM sample (Supplementary material online, Figure S4C).

Treatment with PKA significantly increased the rate of myofibril contraction *k*_ACT_ and decreased *t*_LIN_ in DCM with TTNtv myofibrils but not in donor heart myofibrils (Supplementary material online, Figures S5B and S5C); the increase in *k*_ACT_ was only found in the control sample KN1 with lower phosphorylation level of TnI (Supplementary material online, Figure S4A). PKA treatment significantly increased the rate of the fast relaxation phase *k*_REL_ in donor but not in DCM with TTNtv myofibrils (Supplementary material online, Figure S5D). λ phosphatase treatment did not affect *k*_ACT_ (Supplementary material online, Figure S5A) but significantly prolonged the slow relaxation phase in DCM with TTNtv and healthy donor heart myofibrils (*t*_LIN_; Supplementary material online, Figure S5C). The effect on *k*_REL_ was not significant (Supplementary material online, Figure S5D). The *k*_LIN_ parameter was not significantly different between any group of samples (range 0.24–0.62 s^-1^; Supplementary material online, Figure S4B).

Dephosphorylation of TnI and MyBP-C has a potentially important negative consequence, namely a decrease in the rate of muscle contraction and muscle relaxation (λ phosphatase-treated versus PKA- treated; Supplementary material online, Figures S5B– S5D).

***Force and myosin ATPase in cardiac strips***

The energy cost of force generation provides us with important information about muscle energetics.

Muscle energy consumption is conventionally divided into basal (in relaxed condition) and active states. Measurement of ATP hydrolysis under relaxing conditions can provide us with information about the fraction of myosin heads that are in the super-relaxed state^7^. DCM sample D28 was selected as it was the only one available in a sufficient quantity to perform this experiment.

ATP consumption by myosin was measured in relaxing solution and during isometric contractions at different free Ca^2+^ concentrations. The maximum force produced by thin cardiac strips, prepared from a DCM sample harbouring a truncation mutation in the *TTN* gene, was not significantly decreased compared to healthy donor heart values (Supplementary material online, Figure S7A). The force measured in strips was essentially lower than those of single isolated myofibrils as it was subject to myofibril density dependence and fibrosis. The Ca^2+^ sensitivity of force in D28 was significantly higher than that of the healthy donor heart samples NM and KN1 (Supplementary material online, Figure S7B). Both controls had a very low basal myosin ATPase. We discovered that the DCM sample had significantly elevated ATP consumption in relaxing conditions (5.9 ± 1.4 versus 1.8 ± 1.0 pmol ATP·mm^-3^·s^-1^, two donor hearts; Supplementary material online, Figure S7C). Nevertheless, the energetic cost of tension generation in muscles with TTNtv was comparable to donor heart values (2.54 ± 0.21 versus 2.51 ± 0.14 pmol ATP·mN^-1^·mm^-1^·s^-1^; Supplementary material online, Figure S7D). However, the maximal ATP consumption rate, measured at [Ca^2+^] = 0.1 mol/L, was significantly lower (75.0 ± 5.8 versus 92.0 ± 3.7 pmol ATP·mm^-3^·s^-1^; Supplementary material online, Figure S7E).

The increased basal myosin ATPase activity led to decreased energetic efficiency of the heart even if the tension was not elevated.

**SUPPLEMENTAL FIGURES**

**A**


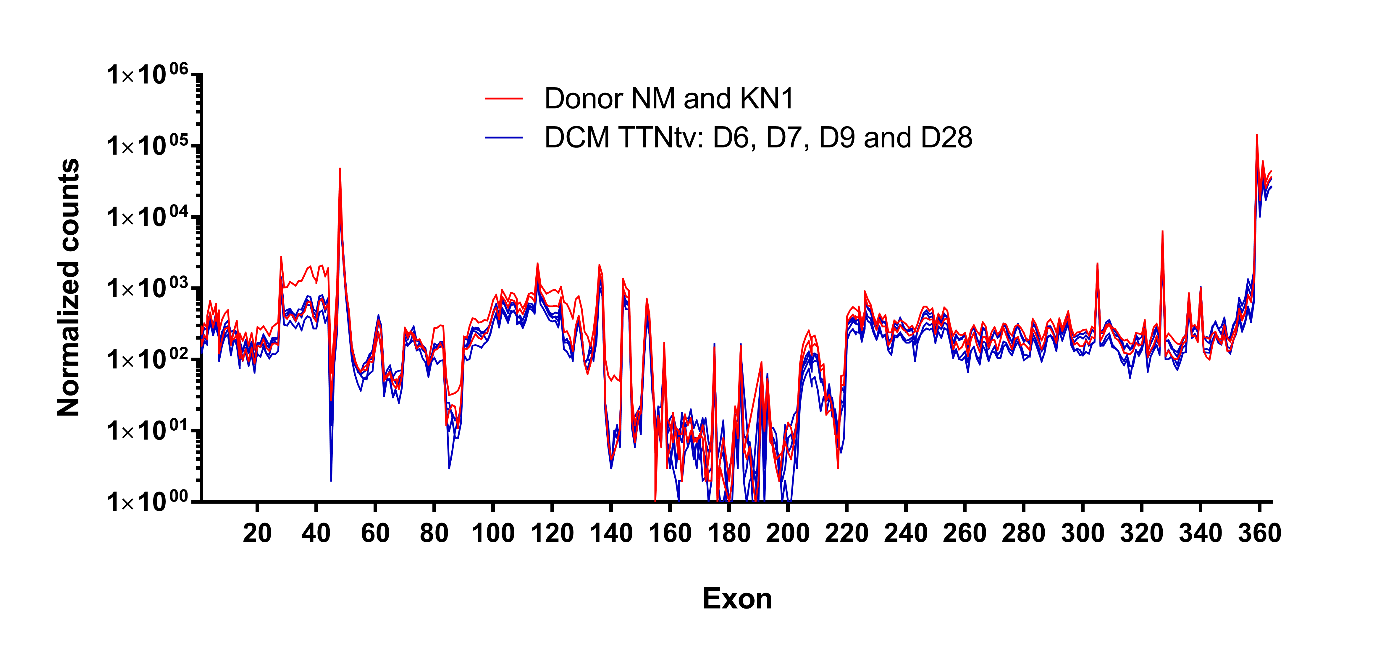


**B**

**
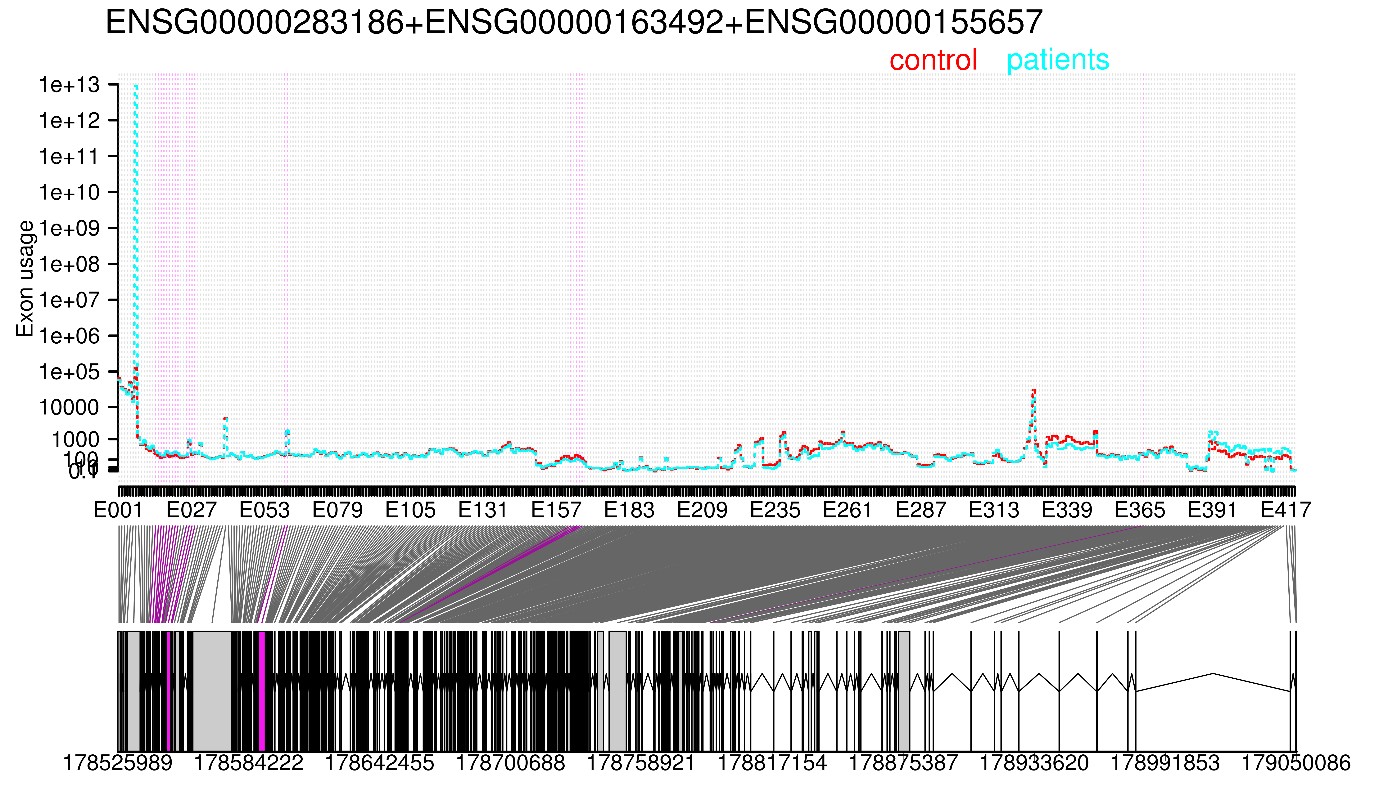
**

**Figure S1. Differential exon usage in the *TTN* gene.** DCM samples with *TTN* mutations (D6, D7, D9 and D28) and healthy donor hearts (KN1 and NM) showed a similar pattern of exon usage of individual samples (**A)** and mean values (**B**). Solid red lines represent donor samples NM and KN1. Blue solid lines represent patient samples D6, D7, D9, and D28. The exons in purple showed significant differential exon usage.


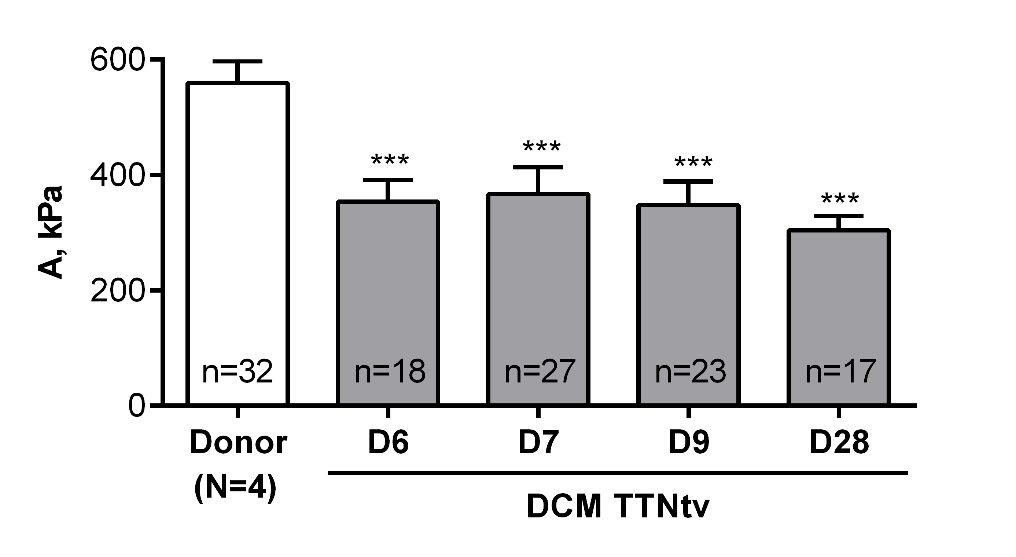


**Figure S2. Myofibril passive stiffness.** The parabolic equation σ(ε) = Aε^2^ was fitted to the stress-strain data, where ε is strain ^1^. The Young’s modulus of D28, defined as E(ε) = 2Aε, was 45.5% lower compared to that of donor heart myofibrils. The Young’s modulus of D6, D7 and D9 samples was determined earlier ^1^. Numbers on bars indicate number of myofibril samples. ***P < 0.001





**Figure S3.** **Myofibril maximal force.** The maximum force of isometric contraction in untreated and treated, with PKA and λ phosphatase, human cardiac myofibrils. Technical replicates from three donors (KN1, NM, KN2) and four patients (D6, D7, D9, D28) were combined, respectively, into two groups: Donors and DCM TTNtv. Numbers on bars indicate number of myofibril samples. Statistical analysis was performed using one-way ANOVA with Fisher's least significant difference test. *P < 0.05, **P < 0.01, ***P < 0.00.

**

**

**Figure S4.** **Kinetic parameters of myofibril contraction and relaxation** **for each sample.** Panels **A**, **B**, **C** and **D** show changes in the rate of force development (*k*_ACT_), rate of slow relaxation phase(*k*_LIN_), duration of slow relaxation phase (t_LIN_) and rate of fast relaxation phase (*k*_REL_). Statistical analysis was performed using one-way ANOVA with Fisher's least significant difference test. #P < 0.05, ##P < 0.01 and ###P < 0.001 versus donor NM. *P < 0.05, **P < 0.01 and ***P < 0.001. Numbers on bars indicate number of myofibril samples. Replicate measurements on the same sample are shown as individual data points.

**
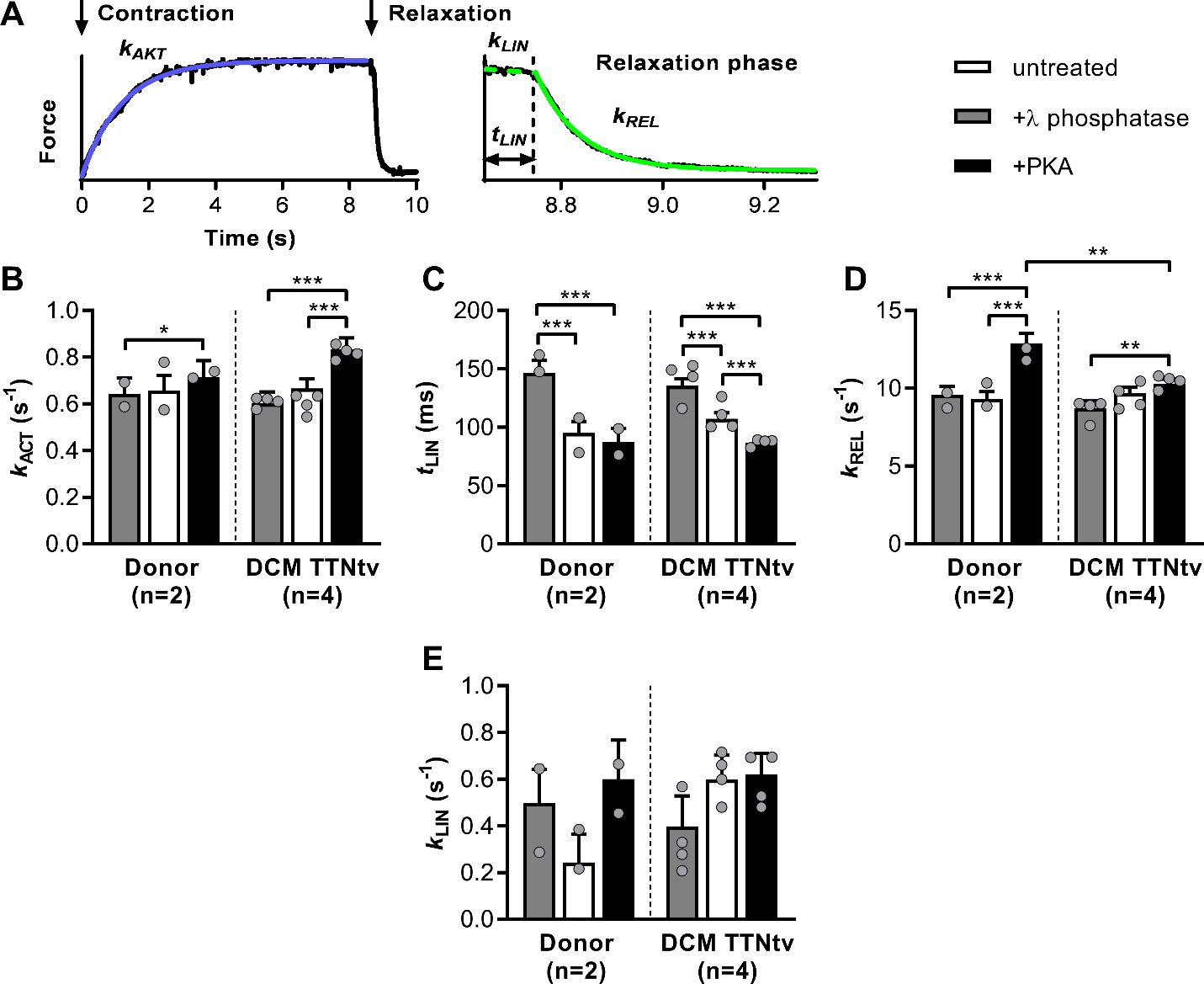
**

**Figure S5.** **Summary of kinetic parameters of myofibril contraction and relaxation.** **A**. Characteristic contractility trace with the relaxation phase on the right side. Cardiac myofibrils were treated with PKA and λ phosphatase to fully phosphorylate and dephosphorylate TnI, respectively. Panels **B**, **C**, **D and E** show changes in the rate of force development (*k*_ACT_), duration of slow relaxation phase (*t*_LIN_), rate of fast relaxation phase (*k*_REL_) and rate of slow relaxation phase (*k*_LIN_) for the combined group of DCMs and healthy donor hearts. Statistical analysis was performed using linear mixed model. Bars show estimated marginal means ± SE. Grey circles represent mean values of individual heart samples. *P < 0.05, **P < 0.01 and ***P < 0.001 versus no treatment and other treatment, or healthy donor. Sarcomere length was 2.0–2.4 µm. Measurements were performed at 17 °C.





**Figure S6.** **Length dependence of the Ca^2+^ sensitivity of force.** The ratios of the EC_50_ values measured at 2.0 µm and 2.4 µm. Bars show means ± SE. Circles represent mean values of individual heart samples. Measurements were performed at 17 °C.

**

**

**Figure S7.** **Force and calcium sensitivity in skinned cardiac strips.** Simultaneous measurement of force production and ATP consumption rate by myosin measured in skinned cardiac strips. ATP consumption was measured, under steady-state condition, in relaxation and during isometric force production. The results gained for DCM heart sample D28 (6-7 muscle strips) were compared to two healthy donor hearts, NM and KN1 (15-17 muscle strips). **A**. Force achieved at maximal Ca^2+^ stimulation. **B**. Calculated EC_50_ values of Ca^2+^ required for a half-maximal force. **C**. Basal ATP activity measured in a relaxing solution containing 10 mM EGTA and no calcium. **D**. ATP consumption normalised per unit of force. **E**. ATP consumption achieved at maximal Ca^2+^ stimulation. Statistical analysis was performed using linear mixed model. Bars show estimated marginal means ± SE. *P < 0.05 and ***P < 0.001. Sarcomere length was 2.2 µm. Measurements were performed at 25 °C.

**SUPPLEMENTAL TABLES**

**Table S1.** Donor characteristics

| Sample | ID | Sex | Age, y | Cause of death |
| --- | --- | --- | --- | --- |
| N12 | 4.104 | F | 59 | Not recorded |
| NA | 5.003 | M | 37 | Intracerebral haemorrhage |
| NB | 5.048 | F | 25 | Subarachnoid haemorrhage |
| NC | 5.054 | M | 27 | Subarachnoid haemorrhage |
| ND* | 5.084 | F | 49 | Hypoxic brain injury |
| NE | 5.086 | M | 29 | Head injury |
| NG | 4.083 | M | 53 | Subarachnoid haemorrhage |
| NH | 5.089 | F | 48 | Subarachnoid haemorrhage |
| NI | 5.090 | F | 42 | Subarachnoid haemorrhage |
| NJ | 5.126 | F | 55 | Subarachnoid haemorrhage |
| NK | 5.128 | M | 45 | Aneurism |
| NL | 5.131 | F | 42 | Hypoxic brain injury |
| NM | 5.138 | M | 23 | Subarachnoid haemorrhage |
| NN | 6.008 | M | 40 | Stroke |
| NS | 7.080 | F | 55 | Subarachnoid haemorrhage |
| KN1 | 24713 | F | 47 | Head trauma |
| KN2 | CF462 | F | 62 | Subarachnoid haemorrhage |

*slight coronary artery disease

**Table S2.** Sample sequencing statistics.

| LV Sample | ID | Sex | Age, y | Total Reads | Total Mapped Reads | Unique Mapped Reads |
| --- | --- | --- | --- | --- | --- | --- |
| Healthy donor |  |  |  |  |  |  |
| NM | 5.138 | M | 23 | 72392465 | 59393463 | 59210756 |
| KN1 | 24713 | F | 47 | 67740279 | 57741164 | 57553100 |
|  | BC90C | F | 39 | 37073460 | 32826738 | 32755415 |
|  | 4B3FA | F | 37 | 29159070 | 25373160 | 25333410 |
|  | D612E | M | 26 | 33129328 | 28028943 | 27974842 |
|  | D0F54 | M | 23 | 34326570 | 30243189 | 30178949 |
| DCM TTNtv |  |  |  |  |  |  |
| D6 | 4.100 | M | 22 | 65384698 | 54043557 | 53896886 |
| D7 | 4.125 | M | 37 | 58439646 | 48545133 | 48394264 |
| D9 | 2.029 | F | 22 | 59706352 | 48981572 | 48854151 |
| D28 | 3.133 | F | 60 | 77503323 | 63510915 | 63330196 |

**Table S3.** Differential *TTN* exon usage results.

Healthy donors: KN1 and NM; DCM patients with TTNtv: D6, D7, D9 and D28.

Ensemble Gene IDs: ENSG00000283186+ENSG00000163492+ENSG00000155657

| Feature ID | Exon Base Mean | Dispersion | P  Value | Adjusted  *P* Value | Start | End | Width | Donors | Patients  (DCM TTNtv) | log2 Fold Change  (patients vs control) |
| --- | --- | --- | --- | --- | --- | --- | --- | --- | --- | --- |
| E001 | 5.997877e+04 | 0.002 | 0.849 | 0.988 | 178525989 | 178527011 | 1023 | 135.889 | 133.237 | -0.028 |
| E002 | 3.441914e+04 | 0.001 | 0.623 | 0.960 | 178527012 | 178527307 | 296 | 123.003 | 120.923 | -0.025 |
| E003 | 3.046891e+04 | 0.001 | 0.341 | 0.885 | 178527446 | 178527748 | 303 | 120.062 | 118.301 | -0.021 |
| E004 | 2.352220e+04 | 0.001 | 0.981 | 0.999 | 178528274 | 178528427 | 154 | 114.686 | 112.222 | -0.031 |
| E005 | 4.489382e+04 | 0.000 | 0.447 | 0.922 | 178528528 | 178529219 | 692 | 129.505 | 126.585 | -0.033 |
| E006 | 1.512014e+04 | 0.001 | 0.044 | 0.490 | 178529960 | 178530116 | 157 | 105.923 | 101.629 | -0.060 |
| E007 | 1.089974e+05 | 0.007 | 0.898 | 0.993 | 178530241 | 178535849 | 5609 | 149.380 | 527.534 | 1.820 |
| E008 | 1.143585e+03 | 0.030 | 0.674 | 0.968 | 178535982 | 178536575 | 594 | 49.207 | 49.776 | 0.017 |
| E009 | 6.241526e+02 | 0.038 | 0.513 | 0.938 | 178536938 | 178537243 | 306 | 38.125 | 39.881 | 0.065 |
| E010 | 8.146014e+02 | 0.028 | 0.157 | 0.746 | 178537342 | 178537917 | 576 | 41.079 | 44.944 | 0.130 |
| E011 | 4.694546e+02 | 0.017 | 0.045 | 0.495 | 178538540 | 178538839 | 300 | 32.526 | 36.156 | 0.153 |
| E012 | 3.736625e+02 | 0.020 | 0.105 | 0.667 | 178538946 | 178539251 | 306 | 29.739 | 32.705 | 0.137 |
| E013 | 5.076187e+02 | 0.011 | 0.016 | 0.318 | 178539382 | 178539966 | 585 | 33.785 | 37.266 | 0.141 |
| E014 | 2.875625e+02 | 0.000 | 0.000 | 0.000 | 178540068 | 178540370 | 303 | 25.781 | 29.406 | 0.190 |
| E015 | 2.296577e+02 | 0.001 | 0.000 | 0.000 | 178541282 | 178541584 | 303 | 22.997 | 26.784 | 0.220 |
| E016 | 1.624540e+02 | 0.001 | 0.000 | 0.000 | 178542264 | 178542563 | 300 | 20.052 | 22.607 | 0.173 |
| E017 | 1.709203e+02 | 0.001 | 0.000 | 0.001 | 178542662 | 178542949 | 288 | 20.588 | 23.016 | 0.161 |
| E018 | 2.910673e+02 | 0.004 | 0.000 | 0.001 | 178543069 | 178543662 | 594 | 25.576 | 29.685 | 0.215 |
| E019 | 2.000666e+02 | 0.007 | 0.000 | 0.028 | 178543834 | 178544115 | 282 | 21.502 | 25.157 | 0.226 |
| E020 | 2.140897e+02 | 0.004 | 0.000 | 0.001 | 178544201 | 178544506 | 306 | 22.015 | 26.054 | 0.243 |
| E021 | 2.625376e+02 | 0.000 | 0.000 | 0.000 | 178545388 | 178545693 | 306 | 23.996 | 28.601 | 0.253 |
| E022 | 2.246764e+02 | 0.001 | 0.000 | 0.000 | 178545820 | 178546116 | 297 | 22.384 | 26.605 | 0.249 |
| E023 | 1.553866e+02 | 0.011 | 0.007 | 0.207 | 178546212 | 178546502 | 291 | 19.679 | 22.086 | 0.167 |
| E024 | 1.616697e+02 | 0.009 | 0.006 | 0.183 | 178546600 | 178546905 | 306 | 20.150 | 22.449 | 0.156 |
| E025 | 1.688162e+02 | 0.001 | 0.000 | 0.021 | 178547003 | 178547305 | 303 | 20.869 | 22.760 | 0.125 |
| E026 | 8.682000e+02 | 0.005 | 0.000 | 0.027 | 178547407 | 178549473 | 2067 | 42.156 | 45.958 | 0.125 |
| E027 | 2.209433e+02 | 0.002 | 0.000 | 0.021 | 178549570 | 178549869 | 300 | 23.700 | 25.618 | 0.112 |
| E028 | 2.569377e+02 | 0.000 | 0.000 | 0.001 | 178549986 | 178550273 | 288 | 25.378 | 27.417 | 0.112 |
| E029 | 2.319015e+02 | 0.001 | 0.013 | 0.284 | 178550967 | 178551260 | 294 | 24.971 | 25.792 | 0.047 |
| E030 | 6.850457e+02 | 0.004 | 0.031 | 0.423 | 178551630 | 178553396 | 1767 | 40.001 | 41.070 | 0.038 |
| E031 | 1.895346e+02 | 0.003 | 0.018 | 0.336 | 178553502 | 178553807 | 306 | 22.523 | 23.815 | 0.080 |
| E032 | 1.685560e+02 | 0.001 | 0.019 | 0.341 | 178553914 | 178554216 | 303 | 21.617 | 22.317 | 0.046 |
| E033 | 1.319998e+02 | 0.001 | 0.039 | 0.467 | 178554453 | 178554752 | 300 | 19.210 | 19.922 | 0.053 |
| E034 | 1.095243e+02 | 0.005 | 0.924 | 0.995 | 178554865 | 178555152 | 288 | 18.713 | 17.600 | -0.088 |
| E035 | 1.222275e+02 | 0.007 | 0.561 | 0.948 | 178556848 | 178557144 | 297 | 19.305 | 18.783 | -0.040 |
| E036 | 1.408002e+02 | 0.005 | 0.324 | 0.878 | 178557253 | 178557555 | 303 | 20.425 | 20.198 | -0.016 |
| E037 | 1.609210e+02 | 0.008 | 0.345 | 0.887 | 178557648 | 178558235 | 588 | 21.692 | 21.591 | -0.007 |
| E038 | 1.463416e+02 | 0.008 | 0.102 | 0.660 | 178558341 | 178558637 | 297 | 20.155 | 21.009 | 0.060 |
| E039 | 4.531463e+03 | 0.007 | 0.260 | 0.840 | 178559311 | 178576416 | 17106 | 77.285 | 76.785 | -0.009 |
| E040 | 1.737399e+02 | 0.004 | 0.226 | 0.815 | 178576529 | 178576831 | 303 | 22.350 | 22.420 | 0.005 |
| E041 | 2.374668e+02 | 0.004 | 0.167 | 0.758 | 178576923 | 178577510 | 588 | 25.710 | 25.951 | 0.013 |
| E042 | 1.945537e+02 | 0.009 | 0.401 | 0.908 | 178577602 | 178577898 | 297 | 23.781 | 23.515 | -0.016 |
| E043 | 1.565147e+02 | 0.004 | 0.035 | 0.448 | 178577988 | 178578185 | 198 | 20.603 | 21.724 | 0.076 |
| E044 | 1.090365e+02 | 0.008 | 0.163 | 0.754 | 178578611 | 178578715 | 105 | 17.535 | 18.305 | 0.062 |
| E045 | 2.777411e+02 | 0.005 | 0.247 | 0.832 | 178578806 | 178579393 | 588 | 27.653 | 27.758 | 0.006 |
| E046 | 1.748105e+02 | 0.007 | 0.329 | 0.879 | 178579561 | 178579848 | 288 | 22.376 | 22.466 | 0.006 |
| E047 | 1.640884e+02 | 0.003 | 0.723 | 0.975 | 178579939 | 178580229 | 291 | 22.270 | 21.509 | -0.050 |
| E048 | 1.835795e+02 | 0.001 | 0.076 | 0.603 | 178580322 | 178580609 | 288 | 22.736 | 23.128 | 0.025 |
| E049 | 1.282089e+02 | 0.005 | 0.607 | 0.958 | 178581499 | 178581804 | 306 | 19.664 | 19.345 | -0.024 |
| E050 | 1.147093e+02 | 0.019 | 0.744 | 0.977 | 178581906 | 178582208 | 303 | 19.374 | 17.982 | -0.108 |
| E051 | 1.409767e+02 | 0.001 | 0.082 | 0.618 | 178582296 | 178582592 | 297 | 20.076 | 20.433 | 0.025 |
| E052 | 1.285311e+02 | 0.002 | 0.832 | 0.987 | 178582940 | 178583227 | 288 | 19.948 | 19.160 | -0.058 |
| E053 | 1.546893e+02 | 0.009 | 0.635 | 0.962 | 178583607 | 178583906 | 300 | 21.445 | 21.171 | -0.019 |
| E054 | 1.832807e+02 | 0.001 | 0.167 | 0.759 | 178584276 | 178584578 | 303 | 23.007 | 22.976 | -0.002 |
| E055 | 2.017876e+02 | 0.001 | 0.196 | 0.789 | 178584669 | 178584968 | 300 | 24.133 | 23.978 | -0.009 |
| E056 | 2.254896e+02 | 0.004 | 0.628 | 0.961 | 178585072 | 178585347 | 276 | 25.655 | 25.131 | -0.030 |
| E057 | 2.908326e+02 | 0.004 | 0.039 | 0.470 | 178586505 | 178586807 | 303 | 27.533 | 28.830 | 0.066 |
| E058 | 2.658651e+02 | 0.003 | 0.088 | 0.632 | 178587118 | 178587417 | 300 | 26.861 | 27.486 | 0.033 |
| E059 | 2.136741e+02 | 0.001 | 0.013 | 0.280 | 178587516 | 178587800 | 285 | 24.106 | 24.990 | 0.052 |
| E060 | 2.014703e+02 | 0.000 | 0.001 | 0.049 | 178587899 | 178588219 | 321 | 23.127 | 24.456 | 0.081 |
| E061 | 1.872441e+03 | 0.001 | 0.000 | 0.004 | 178588538 | 178591504 | 2967 | 57.926 | 59.275 | 0.033 |
| E062 | 2.165845e+02 | 0.000 | 0.002 | 0.095 | 178591599 | 178591892 | 294 | 24.071 | 25.109 | 0.061 |
| E063 | 2.238522e+02 | 0.001 | 0.028 | 0.404 | 178591978 | 178592277 | 300 | 24.838 | 25.333 | 0.028 |
| E064 | 1.798092e+02 | 0.005 | 0.061 | 0.555 | 178592379 | 178592660 | 282 | 22.138 | 23.110 | 0.062 |
| E065 | 2.049933e+02 | 0.010 | 0.257 | 0.838 | 178592775 | 178593083 | 309 | 23.912 | 24.354 | 0.026 |
| E066 | 1.775814e+02 | 0.011 | 0.687 | 0.970 | 178593173 | 178593475 | 303 | 23.076 | 22.426 | -0.041 |
| E067 | 1.804278e+02 | 0.008 | 0.235 | 0.823 | 178593568 | 178593867 | 300 | 22.565 | 22.972 | 0.026 |
| E068 | 1.830788e+02 | 0.001 | 0.121 | 0.696 | 178593961 | 178594242 | 282 | 22.861 | 22.925 | 0.004 |
| E069 | 1.707845e+02 | 0.002 | 0.076 | 0.602 | 178594344 | 178594646 | 303 | 21.896 | 22.335 | 0.029 |
| E070 | 2.221215e+02 | 0.007 | 0.289 | 0.859 | 178595507 | 178595809 | 303 | 24.923 | 25.157 | 0.013 |
| E071 | 3.812782e+02 | 0.007 | 0.257 | 0.838 | 178597538 | 178597819 | 282 | 31.712 | 31.959 | 0.011 |
| E072 | 3.097132e+02 | 0.005 | 0.167 | 0.758 | 178597908 | 178598058 | 151 | 28.749 | 29.293 | 0.027 |
| E073 | 2.406993e+02 | 0.004 | 0.089 | 0.633 | 178598506 | 178598654 | 149 | 25.514 | 26.268 | 0.042 |
| E074 | 3.248061e+02 | 0.004 | 0.216 | 0.808 | 178598748 | 178599062 | 315 | 29.599 | 29.799 | 0.010 |
| E075 | 1.758323e+02 | 0.006 | 0.720 | 0.974 | 178599146 | 178599445 | 300 | 22.957 | 22.223 | -0.047 |
| E076 | 2.315483e+02 | 0.001 | 0.258 | 0.839 | 178599554 | 178599850 | 297 | 25.713 | 25.334 | -0.021 |
| E077 | 2.761712e+02 | 0.000 | 0.188 | 0.782 | 178600854 | 178601171 | 318 | 27.820 | 27.461 | -0.019 |
| E078 | 2.621504e+02 | 0.000 | 0.198 | 0.792 | 178601265 | 178601564 | 300 | 27.206 | 26.877 | -0.018 |
| E079 | 1.621766e+02 | 0.000 | 0.353 | 0.890 | 178601658 | 178601787 | 130 | 22.699 | 21.015 | -0.111 |
| E080 | 1.289542e+02 | 0.001 | 0.401 | 0.908 | 178601882 | 178601914 | 33 | 20.406 | 18.873 | -0.113 |
| E081 | 1.801090e+02 | 0.000 | 0.349 | 0.888 | 178602002 | 178602150 | 149 | 23.006 | 22.611 | -0.025 |
| E082 | 2.437824e+02 | 0.001 | 0.078 | 0.609 | 178602282 | 178602590 | 309 | 26.011 | 26.253 | 0.013 |
| E083 | 2.831221e+02 | 0.001 | 0.047 | 0.505 | 178603876 | 178604305 | 430 | 27.874 | 28.006 | 0.007 |
| E084 | 1.468658e+02 | 0.001 | 0.069 | 0.582 | 178604708 | 178604898 | 191 | 20.467 | 20.805 | 0.024 |
| E085 | 1.543562e+02 | 0.003 | 0.080 | 0.614 | 178604987 | 178605295 | 309 | 20.771 | 21.533 | 0.052 |
| E086 | 1.625099e+02 | 0.005 | 0.701 | 0.972 | 178605414 | 178605713 | 300 | 22.678 | 21.197 | -0.097 |
| E087 | 1.965976e+02 | 0.006 | 0.169 | 0.762 | 178607021 | 178607314 | 294 | 23.565 | 23.798 | 0.014 |
| E088 | 2.497240e+02 | 0.003 | 0.003 | 0.136 | 178607401 | 178607685 | 285 | 25.378 | 27.064 | 0.093 |
| E089 | 2.150279e+02 | 0.001 | 0.122 | 0.699 | 178607785 | 178608081 | 297 | 24.675 | 24.706 | 0.002 |
| E090 | 1.543905e+02 | 0.004 | 0.482 | 0.931 | 178608178 | 178608477 | 300 | 21.420 | 20.998 | -0.029 |
| E091 | 1.799086e+02 | 0.003 | 0.492 | 0.933 | 178608606 | 178608908 | 303 | 23.013 | 22.573 | -0.028 |
| E092 | 2.374876e+02 | 0.003 | 0.144 | 0.731 | 178609208 | 178609570 | 363 | 25.737 | 25.980 | 0.014 |
| E093 | 2.543999e+02 | 0.011 | 0.830 | 0.986 | 178609684 | 178609986 | 303 | 27.148 | 26.500 | -0.035 |
| E094 | 1.856163e+02 | 0.012 | 0.950 | 0.997 | 178610090 | 178610389 | 300 | 23.722 | 22.830 | -0.055 |
| E095 | 1.632062e+02 | 0.011 | 0.281 | 0.854 | 178610993 | 178611271 | 279 | 21.563 | 21.941 | 0.025 |
| E096 | 2.058781e+02 | 0.007 | 0.479 | 0.931 | 178611372 | 178611677 | 306 | 24.488 | 24.150 | -0.020 |
| E097 | 1.787900e+02 | 0.001 | 0.823 | 0.986 | 178611758 | 178611954 | 197 | 23.234 | 22.347 | -0.056 |
| E098 | 1.344309e+02 | 0.001 | 0.861 | 0.989 | 178612057 | 178612162 | 106 | 20.358 | 19.584 | -0.056 |
| E099 | 2.179362e+02 | 0.007 | 0.447 | 0.922 | 178612277 | 178612576 | 300 | 25.021 | 24.844 | -0.010 |
| E100 | 2.370252e+02 | 0.006 | 0.394 | 0.906 | 178612773 | 178613072 | 300 | 26.059 | 25.737 | -0.018 |
| E101 | 1.420748e+02 | 0.010 | 0.856 | 0.989 | 178613161 | 178613276 | 116 | 21.350 | 19.870 | -0.104 |
| E102 | 1.690287e+02 | 0.009 | 0.956 | 0.997 | 178613751 | 178613937 | 187 | 22.953 | 21.710 | -0.080 |
| E103 | 2.315128e+02 | 0.009 | 0.677 | 0.969 | 178614052 | 178614348 | 297 | 25.889 | 25.461 | -0.024 |
| E104 | 1.975666e+02 | 0.007 | 0.389 | 0.904 | 178614466 | 178614753 | 288 | 23.824 | 23.843 | 0.001 |
| E105 | 1.189212e+02 | 0.008 | 0.683 | 0.970 | 178614847 | 178614968 | 122 | 19.113 | 18.632 | -0.037 |
| E106 | 1.820413e+02 | 0.009 | 0.670 | 0.967 | 178615307 | 178615484 | 178 | 23.197 | 22.805 | -0.025 |
| E107 | 1.613358e+02 | 0.009 | 0.490 | 0.933 | 178615641 | 178615788 | 148 | 21.794 | 21.650 | -0.010 |
| E108 | 1.564775e+02 | 0.009 | 0.604 | 0.957 | 178616479 | 178616630 | 152 | 21.692 | 21.209 | -0.032 |
| E109 | 1.893262e+02 | 0.002 | 0.196 | 0.790 | 178616729 | 178617013 | 285 | 23.327 | 23.283 | -0.003 |
| E110 | 1.491232e+02 | 0.008 | 0.910 | 0.993 | 178617120 | 178617234 | 115 | 21.464 | 20.466 | -0.069 |
| E111 | 2.243509e+02 | 0.003 | 0.821 | 0.986 | 178617325 | 178617512 | 188 | 25.746 | 24.799 | -0.054 |
| E112 | 3.686955e+02 | 0.001 | 0.326 | 0.879 | 178617779 | 178618081 | 303 | 31.834 | 31.143 | -0.032 |
| E113 | 3.935114e+02 | 0.002 | 0.199 | 0.793 | 178618189 | 178618491 | 303 | 32.491 | 32.193 | -0.013 |
| E114 | 3.006701e+02 | 0.005 | 0.216 | 0.808 | 178618584 | 178618853 | 270 | 28.545 | 28.870 | 0.016 |
| E115 | 3.536039e+02 | 0.003 | 0.078 | 0.608 | 178619621 | 178619887 | 267 | 30.561 | 31.039 | 0.022 |
| E116 | 2.351855e+02 | 0.000 | 0.104 | 0.665 | 178619988 | 178620112 | 125 | 25.725 | 25.694 | -0.002 |
| E117 | 3.892887e+02 | 0.003 | 0.211 | 0.803 | 178620217 | 178620625 | 409 | 32.282 | 32.090 | -0.009 |
| E118 | 3.711331e+02 | 0.002 | 0.206 | 0.799 | 178620715 | 178620993 | 279 | 31.597 | 31.446 | -0.007 |
| E119 | 4.086449e+02 | 0.005 | 0.305 | 0.869 | 178621102 | 178621368 | 267 | 33.033 | 32.775 | -0.011 |
| E120 | 3.880347e+02 | 0.004 | 0.834 | 0.987 | 178621475 | 178621741 | 267 | 32.991 | 31.663 | -0.059 |
| E121 | 3.113861e+02 | 0.000 | 0.586 | 0.953 | 178621840 | 178622008 | 169 | 30.185 | 28.513 | -0.082 |
| E122 | 2.403739e+02 | 0.000 | 0.462 | 0.926 | 178622670 | 178622767 | 98 | 26.376 | 25.708 | -0.037 |
| E123 | 1.839498e+02 | 0.014 | 0.144 | 0.730 | 178624465 | 178624731 | 267 | 25.111 | 21.740 | -0.208 |
| E124 | 2.403846e+02 | 0.000 | 0.005 | 0.164 | 178625273 | 178625396 | 124 | 25.417 | 26.216 | 0.045 |
| E125 | 2.279144e+02 | 0.000 | 0.004 | 0.143 | 178629301 | 178629443 | 143 | 24.726 | 25.660 | 0.053 |
| E126 | 2.356661e+02 | 0.000 | 0.599 | 0.956 | 178630241 | 178630367 | 127 | 26.248 | 25.442 | -0.045 |
| E127 | 2.643118e+02 | 0.000 | 0.084 | 0.624 | 178630804 | 178630943 | 140 | 27.100 | 27.109 | 0.000 |
| E128 | 3.040000e+02 | 0.005 | 0.089 | 0.635 | 178631034 | 178631300 | 267 | 28.269 | 29.222 | 0.048 |
| E129 | 2.598093e+02 | 0.001 | 0.016 | 0.319 | 178632147 | 178632413 | 267 | 26.418 | 27.099 | 0.037 |
| E130 | 2.781254e+02 | 0.001 | 0.083 | 0.621 | 178632526 | 178632792 | 267 | 27.613 | 27.793 | 0.009 |
| E131 | 1.778333e+02 | 0.001 | 0.749 | 0.977 | 178632918 | 178633044 | 127 | 23.144 | 22.331 | -0.052 |
| E132 | 1.729857e+02 | 0.001 | 0.283 | 0.855 | 178633187 | 178633326 | 140 | 22.460 | 22.178 | -0.018 |
| E133 | 3.007107e+02 | 0.000 | 0.349 | 0.888 | 178633413 | 178633676 | 264 | 29.122 | 28.464 | -0.033 |
| E134 | 2.893019e+02 | 0.002 | 0.161 | 0.751 | 178633817 | 178634083 | 267 | 28.205 | 28.304 | 0.005 |
| E135 | 3.548121e+02 | 0.001 | 0.033 | 0.435 | 178634366 | 178634629 | 264 | 30.658 | 31.013 | 0.017 |
| E136 | 2.489921e+02 | 0.000 | 0.249 | 0.832 | 178634723 | 178634849 | 127 | 26.603 | 26.216 | -0.021 |
| E137 | 3.194770e+02 | 0.003 | 0.693 | 0.971 | 178635165 | 178635304 | 140 | 30.564 | 28.903 | -0.081 |
| E138 | 4.661281e+02 | 0.004 | 0.731 | 0.976 | 178635440 | 178635715 | 276 | 35.896 | 34.084 | -0.075 |
| E139 | 5.115225e+02 | 0.000 | 0.200 | 0.793 | 178635963 | 178636241 | 279 | 37.537 | 35.166 | -0.094 |
| E140 | 6.703373e+02 | 0.001 | 0.271 | 0.847 | 178636398 | 178636751 | 354 | 41.871 | 39.384 | -0.088 |
| E141 | 3.639806e+02 | 0.001 | 0.673 | 0.968 | 178636752 | 178636799 | 48 | 32.255 | 30.592 | -0.076 |
| E142 | 2.610993e+02 | 0.001 | 0.034 | 0.443 | 178637369 | 178637419 | 51 | 26.775 | 27.081 | 0.016 |
| E143 | 4.257445e+02 | 0.003 | 0.812 | 0.985 | 178639699 | 178639771 | 73 | 34.172 | 32.974 | -0.051 |
| E144 | 3.503534e+02 | 0.002 | 0.996 | 1.000 | 178639772 | 178639788 | 17 | 31.574 | 30.208 | -0.064 |
| E145 | 3.644683e+02 | 0.003 | 0.523 | 0.940 | 178640048 | 178640076 | 29 | 32.602 | 30.440 | -0.099 |
| E146 | 3.485647e+02 | 0.006 | 0.515 | 0.938 | 178640077 | 178640110 | 34 | 32.192 | 29.748 | -0.114 |
| E147 | 3.883970e+02 | 0.003 | 0.462 | 0.926 | 178640541 | 178640630 | 90 | 33.604 | 31.231 | -0.106 |
| E148 | 3.471485e+02 | 0.000 | 0.312 | 0.872 | 178641241 | 178641315 | 75 | 31.832 | 29.778 | -0.096 |
| E149 | 2.920585e+02 | 0.003 | 0.453 | 0.924 | 178642237 | 178642317 | 81 | 29.649 | 27.582 | -0.104 |
| E150 | 3.205867e+01 | 0.095 | 0.150 | 0.738 | 178644548 | 178644616 | 69 | 11.780 | 8.741 | -0.431 |
| E151 | 3.176777e+01 | 0.184 | 0.229 | 0.818 | 178645920 | 178646030 | 111 | 11.676 | 8.749 | -0.416 |
| E152 | 7.072812e+00 | 0.008 | 0.071 | 0.588 | 178646485 | 178646559 | 75 | 3.866 | 5.055 | 0.387 |
| E153 | 1.429553e+01 | 0.005 | 0.002 | 0.114 | 178647064 | 178647144 | 81 | 5.202 | 7.307 | 0.490 |
| E154 | 2.176639e+01 | 0.034 | 0.257 | 0.838 | 178647381 | 178647464 | 84 | 7.541 | 8.498 | 0.172 |
| E155 | 3.215791e+01 | 0.032 | 0.024 | 0.381 | 178649248 | 178649331 | 84 | 8.340 | 10.681 | 0.357 |
| E156 | 2.373764e+01 | 0.003 | 0.034 | 0.441 | 178649554 | 178649631 | 78 | 7.703 | 8.949 | 0.216 |
| E157 | 5.588555e+01 | 0.016 | 0.080 | 0.614 | 178649817 | 178649894 | 78 | 14.685 | 11.958 | -0.296 |
| E158 | 5.335434e+01 | 0.036 | 0.289 | 0.859 | 178650164 | 178650271 | 108 | 14.128 | 11.936 | -0.243 |
| E159 | 1.050057e+02 | 0.039 | 0.188 | 0.782 | 178650751 | 178650834 | 84 | 19.816 | 16.409 | -0.272 |
| E160 | 1.278928e+02 | 0.009 | 0.006 | 0.197 | 178651243 | 178651320 | 78 | 22.118 | 17.691 | -0.322 |
| E161 | 1.168655e+02 | 0.011 | 0.005 | 0.181 | 178651453 | 178651516 | 64 | 21.484 | 16.763 | -0.358 |
| E162 | 8.687420e+01 | 0.003 | 0.000 | 0.015 | 178651517 | 178651520 | 4 | 18.616 | 14.522 | -0.358 |
| E163 | 9.539335e+01 | 0.007 | 0.001 | 0.073 | 178651521 | 178651536 | 16 | 19.586 | 15.114 | -0.374 |
| E164 | 1.413071e+02 | 0.001 | 0.000 | 0.000 | 178651666 | 178651749 | 84 | 23.416 | 18.232 | -0.361 |
| E165 | 1.165138e+02 | 0.001 | 0.000 | 0.000 | 178651884 | 178651967 | 84 | 21.549 | 16.567 | -0.379 |
| E166 | 8.945258e+01 | 0.001 | 0.000 | 0.011 | 178652096 | 178652179 | 84 | 18.660 | 14.817 | -0.333 |
| E167 | 4.996598e+01 | 0.002 | 0.006 | 0.196 | 178652264 | 178652347 | 84 | 13.980 | 11.245 | -0.314 |
| E168 | 1.206037e+01 | 0.048 | 0.979 | 0.999 | 178652458 | 178652541 | 84 | 6.265 | 6.078 | -0.044 |
| E169 | 8.039904e+00 | 0.060 | 0.294 | 0.862 | 178652653 | 178652736 | 84 | 4.361 | 5.364 | 0.299 |
| E170 | 7.000318e+00 | 0.194 | 0.793 | 0.983 | 178652848 | 178652931 | 84 | 4.460 | 4.820 | 0.112 |
| E171 | 7.136184e+00 | 0.194 | 0.887 | 0.992 | 178653041 | 178653124 | 84 | 4.884 | 4.651 | -0.070 |
| E172 | 6.580039e+00 | 0.058 | 0.958 | 0.997 | 178653238 | 178653321 | 84 | 4.653 | 4.588 | -0.020 |
| E173 | 3.187133e+00 | 0.018 | 0.397 | 0.907 | 178653427 | 178653507 | 81 | 3.684 | 2.871 | -0.359 |
| E174 | 2.558276e+00 | 0.030 | 0.032 | 0.430 | 178653621 | 178653701 | 81 | 1.646 | 3.282 | 0.995 |
| E175 | 5.386925e+00 | 0.011 | 0.038 | 0.460 | 178653817 | 178653897 | 81 | 3.083 | 4.542 | 0.559 |
| E176 | 6.992852e+00 | 0.011 | 0.096 | 0.649 | 178654012 | 178654095 | 84 | 3.864 | 4.980 | 0.366 |
| E177 | 1.036155e+01 | 0.049 | 0.548 | 0.945 | 178654208 | 178654291 | 84 | 5.430 | 5.828 | 0.102 |
| E178 | 4.614900e+01 | 0.019 | 0.342 | 0.885 | 178654445 | 178654534 | 90 | 11.477 | 12.187 | 0.087 |
| E179 | 7.307255e+00 | 0.078 | 0.120 | 0.694 | 178654745 | 178654828 | 84 | 5.973 | 4.019 | -0.572 |
| E180 | 6.559699e+01 | 0.032 | 0.997 | 1.000 | 178654912 | 178654995 | 84 | 14.532 | 13.895 | -0.065 |
| E181 | 1.946466e+00 | 0.396 | 0.309 | 0.871 | 178657498 | 178657581 | 84 | 1.628 | 2.930 | 0.848 |
| E182 | 2.745699e-01 | 0.187 | 0.776 |  | 178657687 | 178657767 | 81 |  |  |  |
| E183 | 8.075187e-01 | 0.338 | 0.997 |  | 178657881 | 178657961 | 81 |  |  |  |
| E184 | 4.015229e+00 | 0.170 | 0.247 | 0.831 | 178658077 | 178658157 | 81 | 2.813 | 3.950 | 0.490 |
| E185 | 1.274367e+00 | 0.658 | 0.791 |  | 178658272 | 178658355 | 84 |  |  |  |
| E186 | 3.623442e+00 | 0.206 | 0.533 | 0.942 | 178658468 | 178658551 | 84 | 3.862 | 3.190 | -0.276 |
| E187 | 1.283181e+02 | 0.022 | 0.972 | 0.998 | 178658705 | 178658794 | 90 | 19.925 | 19.245 | -0.050 |
| E188 | 8.397407e+00 | 0.076 | 0.547 | 0.945 | 178659005 | 178659088 | 84 | 5.625 | 4.747 | -0.245 |
| E189 | 1.398553e+01 | 0.004 | 0.475 | 0.929 | 178659172 | 178659255 | 84 | 7.162 | 6.277 | -0.190 |
| E190 | 2.377045e+00 | 0.047 | 0.362 | 0.894 | 178661759 | 178661842 | 84 | 3.309 | 2.395 | -0.466 |
| E191 | 1.243083e+00 | 0.049 | 0.894 |  | 178661948 | 178662028 | 81 |  |  |  |
| E192 | 4.382520e-01 | 0.124 | 0.106 |  | 178662142 | 178662222 | 81 |  |  |  |
| E193 | 6.271584e+00 | 0.153 | 0.340 | 0.884 | 178662338 | 178662418 | 81 | 3.735 | 4.781 | 0.356 |
| E194 | 3.798184e+00 | 0.019 | 0.374 | 0.898 | 178662533 | 178662616 | 84 | 3.084 | 3.634 | 0.237 |
| E195 | 2.207862e+00 | 0.025 | 0.215 | 0.806 | 178662729 | 178662812 | 84 | 2.020 | 2.876 | 0.510 |
| E196 | 1.240708e+02 | 0.025 | 0.883 | 0.991 | 178662966 | 178663055 | 90 | 19.463 | 19.034 | -0.032 |
| E197 | 4.456296e+00 | 0.111 | 0.942 | 0.996 | 178663266 | 178663349 | 84 | 3.824 | 3.741 | -0.032 |
| E198 | 2.891754e+00 | 0.195 | 0.770 | 0.980 | 178663433 | 178663516 | 84 | 2.842 | 3.215 | 0.178 |
| E199 | 5.433797e+00 | 0.278 | 0.523 | 0.940 | 178663627 | 178663710 | 84 | 3.554 | 4.481 | 0.335 |
| E200 | 8.763253e+00 | 0.048 | 0.814 | 0.985 | 178663819 | 178663902 | 84 | 5.225 | 5.272 | 0.013 |
| E201 | 7.621802e+00 | 0.009 | 0.683 | 0.970 | 178664015 | 178664098 | 84 | 4.798 | 4.891 | 0.028 |
| E202 | 7.746726e+00 | 0.024 | 0.343 | 0.886 | 178664460 | 178664537 | 78 | 4.512 | 5.135 | 0.186 |
| E203 | 7.222953e+00 | 0.009 | 0.447 | 0.922 | 178664654 | 178664737 | 84 | 4.508 | 4.888 | 0.117 |
| E204 | 1.256775e+01 | 0.006 | 0.230 | 0.819 | 178664852 | 178664926 | 75 | 5.819 | 6.473 | 0.154 |
| E205 | 9.587297e+00 | 0.007 | 0.242 | 0.829 | 178665377 | 178665460 | 84 | 5.076 | 5.730 | 0.175 |
| E206 | 1.178126e+01 | 0.007 | 0.837 | 0.987 | 178665708 | 178665791 | 84 | 6.158 | 6.011 | -0.035 |
| E207 | 8.235558e+00 | 0.139 | 0.147 | 0.734 | 178666824 | 178666901 | 78 | 3.920 | 5.629 | 0.522 |
| E208 | 7.119431e+00 | 0.021 | 0.873 | 0.990 | 178667236 | 178667307 | 72 | 4.803 | 4.710 | -0.028 |
| E209 | 5.874160e+00 | 0.025 | 0.886 | 0.992 | 178667308 | 178667319 | 12 | 4.371 | 4.251 | -0.040 |
| E210 | 6.606436e+00 | 0.067 | 0.325 | 0.878 | 178667442 | 178667525 | 84 | 5.221 | 4.077 | -0.357 |
| E211 | 1.045182e+01 | 0.125 | 0.924 | 0.995 | 178667638 | 178667721 | 84 | 5.755 | 5.761 | 0.002 |
| E212 | 8.504366e+00 | 0.044 | 0.586 | 0.953 | 178669373 | 178669447 | 75 | 4.964 | 5.276 | 0.088 |
| E213 | 8.556766e+00 | 0.014 | 0.667 | 0.967 | 178669592 | 178669624 | 33 | 5.090 | 5.135 | 0.013 |
| E214 | 9.571989e+00 | 0.063 | 0.492 | 0.933 | 178669625 | 178669675 | 51 | 5.278 | 5.474 | 0.053 |
| E215 | 1.251318e+02 | 0.001 | 0.773 | 0.981 | 178670218 | 178670295 | 78 | 19.584 | 18.870 | -0.054 |
| E216 | 1.016073e+01 | 0.073 | 0.203 | 0.796 | 178671090 | 178671170 | 81 | 4.683 | 6.056 | 0.371 |
| E217 | 1.577729e+01 | 0.004 | 0.179 | 0.774 | 178671971 | 178672267 | 297 | 6.573 | 7.296 | 0.151 |
| E218 | 6.384711e+00 | 0.062 | 0.013 | 0.284 | 178672407 | 178672418 | 12 | 2.870 | 5.081 | 0.824 |
| E219 | 1.179355e+01 | 0.015 | 0.004 | 0.158 | 178672419 | 178672481 | 63 | 4.516 | 6.677 | 0.564 |
| E220 | 5.868807e+01 | 0.006 | 0.443 | 0.921 | 178672635 | 178672703 | 69 | 14.168 | 12.837 | -0.142 |
| E221 | 3.027997e+02 | 0.004 | 0.268 | 0.845 | 178673633 | 178673710 | 78 | 30.475 | 27.777 | -0.134 |
| E222 | 5.029982e+02 | 0.004 | 0.190 | 0.784 | 178674314 | 178674409 | 96 | 37.855 | 34.627 | -0.129 |
| E223 | 1.258331e+02 | 0.010 | 0.253 | 0.835 | 178675039 | 178675113 | 75 | 18.864 | 19.502 | 0.048 |
| E224 | 1.628011e+01 | 0.037 | 0.932 | 0.995 | 178675671 | 178675754 | 84 | 7.162 | 7.017 | -0.030 |
| E225 | 1.632442e+01 | 0.003 | 0.144 | 0.730 | 178675921 | 178675995 | 75 | 6.576 | 7.358 | 0.162 |
| E226 | 9.498227e+00 | 0.011 | 0.340 | 0.885 | 178677201 | 178677287 | 87 | 5.079 | 5.595 | 0.140 |
| E227 | 1.964950e+01 | 0.032 | 0.451 | 0.923 | 178677621 | 178677917 | 297 | 7.582 | 7.927 | 0.064 |
| E228 | 6.656588e+02 | 0.004 | 0.565 | 0.949 | 178678125 | 178678208 | 84 | 41.854 | 39.244 | -0.093 |
| E229 | 7.007759e+02 | 0.004 | 0.301 | 0.867 | 178678414 | 178678497 | 84 | 43.145 | 39.782 | -0.117 |
| E230 | 9.271787e+02 | 0.007 | 0.432 | 0.918 | 178678747 | 178678830 | 84 | 48.011 | 44.451 | -0.111 |
| E231 | 1.684605e+01 | 0.112 | 0.168 | 0.760 | 178679339 | 178679416 | 78 | 9.285 | 5.696 | -0.705 |
| E232 | 1.629424e+01 | 0.153 | 0.287 | 0.858 | 178679599 | 178679682 | 84 | 9.267 | 5.485 | -0.757 |
| E233 | 1.621434e+01 | 0.222 | 0.321 | 0.876 | 178679894 | 178680055 | 162 | 9.690 | 5.051 | -0.940 |
| E234 | 1.138304e+01 | 0.259 | 0.066 | 0.572 | 178680254 | 178680331 | 78 | 8.926 | 3.164 | -1.496 |
| E235 | 1.754821e+01 | 0.171 | 0.280 | 0.853 | 178681079 | 178681171 | 93 | 9.805 | 5.530 | -0.826 |
| E236 | 3.497661e+01 | 0.076 | 0.104 | 0.664 | 178681376 | 178681450 | 75 | 13.208 | 8.293 | -0.672 |
| E237 | 1.086571e+03 | 0.008 | 0.453 | 0.923 | 178681661 | 178681738 | 78 | 50.732 | 47.319 | -0.100 |
| E238 | 1.478224e+03 | 0.010 | 0.442 | 0.921 | 178682697 | 178682903 | 207 | 56.656 | 52.849 | -0.100 |
| E239 | 3.265162e+02 | 0.015 | 0.046 | 0.501 | 178683211 | 178683291 | 81 | 33.257 | 27.594 | -0.269 |
| E240 | 1.434226e+02 | 0.035 | 0.236 | 0.824 | 178683999 | 178684082 | 84 | 22.924 | 18.940 | -0.275 |
| E241 | 1.251265e+02 | 0.027 | 0.113 | 0.681 | 178684330 | 178684413 | 84 | 17.909 | 19.893 | 0.152 |
| E242 | 1.124645e+02 | 0.076 | 0.813 | 0.985 | 178684666 | 178684749 | 84 | 19.345 | 17.638 | -0.133 |
| E243 | 1.157639e+02 | 0.043 | 0.393 | 0.906 | 178684906 | 178684989 | 84 | 21.071 | 16.873 | -0.321 |
| E244 | 2.658962e+02 | 0.026 | 0.313 | 0.872 | 178685253 | 178685330 | 78 | 30.284 | 25.263 | -0.262 |
| E245 | 4.556632e+02 | 0.015 | 0.548 | 0.945 | 178685518 | 178685598 | 81 | 36.782 | 32.971 | -0.158 |
| E246 | 3.433549e+02 | 0.031 | 0.206 | 0.799 | 178688111 | 178688224 | 114 | 34.438 | 27.872 | -0.305 |
| E247 | 1.606301e+02 | 0.042 | 0.128 | 0.708 | 178688677 | 178688778 | 102 | 25.604 | 18.885 | -0.439 |
| E248 | 2.150634e+02 | 0.026 | 0.017 | 0.325 | 178689053 | 178689136 | 84 | 29.464 | 21.416 | -0.460 |
| E249 | 2.572192e+02 | 0.032 | 0.021 | 0.356 | 178689290 | 178689373 | 84 | 32.091 | 23.066 | -0.476 |
| E250 | 2.613017e+02 | 0.036 | 0.019 | 0.345 | 178689515 | 178689595 | 81 | 32.743 | 22.836 | -0.520 |
| E251 | 6.787013e+02 | 0.009 | 0.235 | 0.823 | 178689813 | 178689896 | 84 | 43.194 | 38.913 | -0.151 |
| E252 | 4.842893e+02 | 0.023 | 0.065 | 0.572 | 178692016 | 178692099 | 84 | 39.527 | 32.535 | -0.281 |
| E253 | 5.257450e+02 | 0.020 | 0.075 | 0.600 | 178692497 | 178692580 | 84 | 40.707 | 33.804 | -0.268 |
| E254 | 5.115205e+02 | 0.016 | 0.043 | 0.488 | 178693609 | 178693689 | 81 | 40.404 | 33.296 | -0.279 |
| E255 | 5.238957e+02 | 0.012 | 0.124 | 0.700 | 178693922 | 178694008 | 87 | 39.729 | 34.432 | -0.206 |
| E256 | 5.760985e+02 | 0.006 | 0.664 | 0.967 | 178694599 | 178694676 | 78 | 39.594 | 36.951 | -0.100 |
| E257 | 6.934508e+02 | 0.005 | 0.656 | 0.965 | 178694829 | 178694906 | 78 | 42.488 | 39.940 | -0.089 |
| E258 | 8.105211e+02 | 0.005 | 0.650 | 0.965 | 178695348 | 178695410 | 63 | 45.095 | 42.522 | -0.085 |
| E259 | 1.587229e+03 | 0.006 | 0.646 | 0.964 | 178695865 | 178696269 | 405 | 57.499 | 54.520 | -0.077 |
| E260 | 5.698088e+02 | 0.004 | 0.326 | 0.879 | 178697121 | 178697168 | 48 | 39.616 | 36.626 | -0.113 |
| E261 | 6.381567e+02 | 0.008 | 0.502 | 0.936 | 178698843 | 178698914 | 72 | 41.338 | 38.491 | -0.103 |
| E262 | 6.432387e+02 | 0.008 | 0.598 | 0.956 | 178701120 | 178701203 | 84 | 41.273 | 38.751 | -0.091 |
| E263 | 4.521847e+02 | 0.008 | 0.297 | 0.864 | 178701528 | 178701587 | 60 | 36.277 | 33.099 | -0.132 |
| E264 | 3.658402e+02 | 0.005 | 0.140 | 0.725 | 178702040 | 178702066 | 27 | 33.358 | 30.035 | -0.151 |
| E265 | 4.352788e+02 | 0.003 | 0.072 | 0.591 | 178702168 | 178702245 | 78 | 35.925 | 32.357 | -0.151 |
| E266 | 4.058418e+02 | 0.001 | 0.156 | 0.745 | 178702454 | 178702520 | 67 | 34.438 | 31.631 | -0.123 |
| E267 | 5.131645e+02 | 0.002 | 0.395 | 0.906 | 178702521 | 178702663 | 143 | 37.630 | 35.218 | -0.096 |
| E268 | 6.152718e+02 | 0.001 | 0.689 | 0.970 | 178704147 | 178704407 | 261 | 40.192 | 38.161 | -0.075 |
| E269 | 6.135439e+02 | 0.001 | 0.168 | 0.759 | 178704510 | 178704777 | 268 | 40.694 | 37.770 | -0.108 |
| E270 | 4.639883e+02 | 0.001 | 0.043 | 0.485 | 178704877 | 178704966 | 90 | 36.471 | 33.459 | -0.124 |
| E271 | 5.760896e+02 | 0.001 | 0.108 | 0.672 | 178705174 | 178705357 | 184 | 39.663 | 36.817 | -0.107 |
| E272 | 6.782143e+02 | 0.001 | 0.242 | 0.828 | 178706454 | 178706739 | 286 | 42.153 | 39.472 | -0.095 |
| E273 | 3.759556e+02 | 0.001 | 0.883 | 0.991 | 178706862 | 178706954 | 93 | 32.507 | 31.107 | -0.063 |
| E274 | 5.636579e+02 | 0.002 | 0.657 | 0.965 | 178707526 | 178707813 | 288 | 38.457 | 37.038 | -0.054 |
| E275 | 4.571208e+02 | 0.001 | 0.176 | 0.770 | 178709566 | 178709856 | 291 | 36.095 | 33.344 | -0.114 |
| E276 | 3.146560e+02 | 0.001 | 0.218 | 0.809 | 178710635 | 178710922 | 288 | 30.724 | 28.400 | -0.114 |
| E277 | 2.444441e+02 | 0.002 | 0.960 | 0.997 | 178711062 | 178711349 | 288 | 26.997 | 25.643 | -0.074 |
| E278 | 2.418027e+02 | 0.006 | 0.992 | 1.000 | 178711944 | 178712222 | 279 | 26.993 | 25.493 | -0.082 |
| E279 | 2.275950e+02 | 0.012 | 0.615 | 0.959 | 178712315 | 178712593 | 279 | 25.872 | 25.085 | -0.045 |
| E280 | 2.418746e+02 | 0.010 | 0.224 | 0.814 | 178712697 | 178712975 | 279 | 25.838 | 26.238 | 0.022 |
| E281 | 2.587939e+02 | 0.013 | 0.574 | 0.951 | 178713085 | 178713372 | 288 | 27.287 | 26.685 | -0.032 |
| E282 | 2.551371e+02 | 0.023 | 0.459 | 0.925 | 178713897 | 178714175 | 279 | 26.876 | 26.652 | -0.012 |
| E283 | 1.921822e+02 | 0.025 | 0.720 | 0.974 | 178714292 | 178714573 | 282 | 24.051 | 23.201 | -0.052 |
| E284 | 1.572188e+02 | 0.011 | 0.282 | 0.855 | 178714986 | 178715264 | 279 | 21.074 | 21.574 | 0.034 |
| E285 | 1.646908e+02 | 0.010 | 0.185 | 0.778 | 178715493 | 178715774 | 282 | 21.386 | 22.092 | 0.047 |
| E286 | 2.426179e+01 | 0.012 | 0.073 | 0.595 | 178717095 | 178717382 | 288 | 9.935 | 7.746 | -0.359 |
| E287 | 1.654609e+01 | 0.047 | 0.449 | 0.922 | 178717523 | 178717810 | 288 | 8.162 | 6.482 | -0.332 |
| E288 | 1.684482e+01 | 0.004 | 0.007 | 0.205 | 178717943 | 178718221 | 279 | 8.682 | 6.215 | -0.482 |
| E289 | 1.795144e+01 | 0.006 | 0.040 | 0.473 | 178718322 | 178718600 | 279 | 8.686 | 6.656 | -0.384 |
| E290 | 1.768329e+01 | 0.090 | 0.395 | 0.906 | 178718695 | 178718973 | 279 | 8.373 | 6.848 | -0.290 |
| E291 | 2.656385e+01 | 0.095 | 0.976 | 0.998 | 178719164 | 178719451 | 288 | 9.941 | 8.489 | -0.228 |
| E292 | 1.724499e+02 | 0.025 | 0.828 | 0.986 | 178719554 | 178719832 | 279 | 23.769 | 21.491 | -0.145 |
| E293 | 1.773142e+02 | 0.015 | 0.379 | 0.900 | 178719983 | 178720264 | 282 | 24.527 | 21.473 | -0.192 |
| E294 | 1.606381e+02 | 0.023 | 0.579 | 0.952 | 178720385 | 178720663 | 279 | 23.365 | 20.562 | -0.184 |
| E295 | 1.639714e+02 | 0.017 | 0.827 | 0.986 | 178720921 | 178721202 | 282 | 23.174 | 21.010 | -0.141 |
| E296 | 1.120234e+02 | 0.002 | 0.533 | 0.942 | 178721847 | 178722134 | 288 | 18.412 | 17.986 | -0.034 |
| E297 | 7.693544e+01 | 0.001 | 0.593 | 0.955 | 178722259 | 178722546 | 288 | 15.396 | 15.079 | -0.030 |
| E298 | 1.277640e+02 | 0.017 | 0.182 | 0.776 | 178722659 | 178722937 | 279 | 18.511 | 19.887 | 0.103 |
| E299 | 1.442862e+02 | 0.016 | 0.222 | 0.812 | 178723046 | 178723324 | 279 | 19.781 | 20.954 | 0.083 |
| E300 | 1.722042e+02 | 0.006 | 0.021 | 0.359 | 178723418 | 178723696 | 279 | 21.226 | 22.836 | 0.105 |
| E301 | 1.558814e+02 | 0.006 | 0.073 | 0.593 | 178723856 | 178724143 | 288 | 20.604 | 21.604 | 0.068 |
| E302 | 2.309938e+02 | 0.014 | 0.012 | 0.272 | 178724260 | 178724538 | 279 | 23.256 | 26.710 | 0.200 |
| E303 | 2.202798e+02 | 0.004 | 0.151 | 0.739 | 178725368 | 178725649 | 282 | 24.721 | 25.163 | 0.026 |
| E304 | 1.930263e+02 | 0.000 | 0.102 | 0.660 | 178725768 | 178726046 | 279 | 23.445 | 23.538 | 0.006 |
| E305 | 2.352566e+02 | 0.004 | 0.713 | 0.973 | 178727090 | 178727371 | 282 | 26.188 | 25.511 | -0.038 |
| E306 | 6.099428e+01 | 0.010 | 0.063 | 0.563 | 178727585 | 178727863 | 279 | 12.688 | 14.076 | 0.150 |
| E307 | 4.785187e+01 | 0.029 | 0.280 | 0.853 | 178728110 | 178728397 | 288 | 11.391 | 12.450 | 0.128 |
| E308 | 4.677232e+01 | 0.019 | 0.156 | 0.745 | 178728500 | 178728778 | 279 | 11.119 | 12.425 | 0.160 |
| E309 | 4.359841e+01 | 0.009 | 0.341 | 0.885 | 178728891 | 178729169 | 279 | 11.310 | 11.777 | 0.058 |
| E310 | 6.387256e+01 | 0.001 | 0.016 | 0.318 | 178729288 | 178729566 | 279 | 13.161 | 14.390 | 0.129 |
| E311 | 5.852149e+01 | 0.003 | 0.028 | 0.404 | 178729664 | 178729945 | 282 | 12.562 | 13.764 | 0.132 |
| E312 | 4.871376e+01 | 0.008 | 0.315 | 0.873 | 178730093 | 178730371 | 279 | 11.929 | 12.385 | 0.054 |
| E313 | 2.298789e+02 | 0.049 | 0.088 | 0.631 | 178730505 | 178730792 | 288 | 22.476 | 27.047 | 0.267 |
| E314 | 3.259322e+02 | 0.029 | 0.042 | 0.481 | 178730925 | 178731203 | 279 | 26.845 | 31.339 | 0.223 |
| E315 | 1.954552e+02 | 0.019 | 0.020 | 0.348 | 178731305 | 178731583 | 279 | 21.402 | 24.892 | 0.218 |
| E316 | 1.051850e+02 | 0.016 | 0.051 | 0.518 | 178731693 | 178731971 | 279 | 16.296 | 18.501 | 0.183 |
| E317 | 9.793543e+01 | 0.013 | 0.088 | 0.632 | 178732066 | 178732347 | 282 | 16.088 | 17.670 | 0.135 |
| E318 | 7.095149e+01 | 0.013 | 0.339 | 0.884 | 178732440 | 178732718 | 279 | 14.280 | 14.878 | 0.059 |
| E319 | 6.838938e+01 | 0.007 | 0.314 | 0.873 | 178732834 | 178733121 | 288 | 14.111 | 14.534 | 0.043 |
| E320 | 5.947502e+01 | 0.008 | 0.533 | 0.942 | 178733239 | 178733517 | 279 | 13.422 | 13.439 | 0.002 |
| E321 | 6.758771e+01 | 0.009 | 0.559 | 0.948 | 178733614 | 178733892 | 279 | 14.301 | 14.294 | -0.001 |
| E322 | 9.001819e+01 | 0.017 | 0.353 | 0.890 | 178734328 | 178734606 | 279 | 15.980 | 16.658 | 0.060 |
| E323 | 1.393584e+02 | 0.017 | 0.223 | 0.813 | 178734707 | 178734988 | 282 | 19.428 | 20.628 | 0.086 |
| E324 | 4.174976e+02 | 0.022 | 0.838 | 0.987 | 178735511 | 178736074 | 564 | 33.576 | 32.968 | -0.026 |
| E325 | 9.083901e+02 | 0.011 | 0.023 | 0.373 | 178738082 | 178738360 | 279 | 43.195 | 46.655 | 0.111 |
| E326 | 3.209116e+03 | 0.010 | 0.008 | 0.231 | 178739141 | 178741921 | 2781 | 66.504 | 71.355 | 0.102 |
| E327 | 2.207237e+04 | 0.051 | 0.399 | 0.908 | 178744405 | 178752039 | 7635 | 118.232 | 107.032 | -0.144 |
| E328 | 4.477734e+02 | 0.043 | 0.192 | 0.786 | 178753124 | 178753180 | 57 | 39.654 | 30.362 | -0.385 |
| E329 | 1.962493e+02 | 0.019 | 0.040 | 0.473 | 178753361 | 178754009 | 649 | 27.395 | 21.241 | -0.367 |
| E330 | 1.234761e+02 | 0.008 | 0.167 | 0.758 | 178756222 | 178756797 | 576 | 20.934 | 17.964 | -0.221 |
| E331 | 2.243063e+01 | 0.097 | 0.009 | 0.243 | 178757542 | 178757916 | 375 | 11.347 | 6.019 | -0.915 |
| E332 | 8.228042e+02 | 0.049 | 0.286 | 0.857 | 178758984 | 178759172 | 189 | 49.696 | 39.583 | -0.328 |
| E333 | 6.512360e+02 | 0.045 | 0.218 | 0.809 | 178764177 | 178764302 | 126 | 45.536 | 35.974 | -0.340 |
| E334 | 9.146616e+02 | 0.046 | 0.248 | 0.832 | 178764527 | 178764811 | 285 | 51.561 | 41.358 | -0.318 |
| E335 | 8.840579e+02 | 0.048 | 0.283 | 0.855 | 178766381 | 178766612 | 232 | 50.882 | 40.850 | -0.317 |
| E336 | 5.115537e+02 | 0.052 | 0.295 | 0.863 | 178767759 | 178767825 | 67 | 41.597 | 32.362 | -0.362 |
| E337 | 5.304590e+02 | 0.068 | 0.418 | 0.913 | 178767826 | 178767924 | 99 | 42.225 | 32.847 | -0.362 |
| E338 | 5.842882e+02 | 0.074 | 0.327 | 0.879 | 178768014 | 178768155 | 142 | 44.341 | 33.782 | -0.392 |
| E339 | 8.363111e+02 | 0.056 | 0.182 | 0.776 | 178768673 | 178768933 | 261 | 50.811 | 39.087 | -0.378 |
| E340 | 8.355345e+02 | 0.054 | 0.272 | 0.848 | 178769679 | 178769939 | 261 | 49.982 | 39.825 | -0.328 |
| E341 | 6.819691e+02 | 0.055 | 0.432 | 0.918 | 178770060 | 178770320 | 261 | 45.866 | 37.079 | -0.307 |
| E342 | 5.455781e+02 | 0.047 | 0.207 | 0.800 | 178770412 | 178770675 | 264 | 42.775 | 33.171 | -0.367 |
| E343 | 5.684330e+02 | 0.041 | 0.278 | 0.852 | 178771211 | 178771471 | 261 | 42.838 | 34.289 | -0.321 |
| E344 | 4.777708e+02 | 0.050 | 0.330 | 0.880 | 178773109 | 178773369 | 261 | 40.137 | 31.711 | -0.340 |
| E345 | 5.288396e+02 | 0.038 | 0.242 | 0.828 | 178773462 | 178773725 | 264 | 41.611 | 33.273 | -0.323 |
| E346 | 5.871591e+02 | 0.040 | 0.484 | 0.932 | 178773838 | 178774110 | 273 | 42.468 | 35.501 | -0.259 |
| E347 | 5.134097e+02 | 0.037 | 0.611 | 0.958 | 178774207 | 178774473 | 267 | 39.745 | 33.944 | -0.228 |
| E348 | 5.104161e+02 | 0.029 | 0.368 | 0.896 | 178774921 | 178775202 | 282 | 40.001 | 33.589 | -0.252 |
| E349 | 1.515928e+03 | 0.020 | 0.481 | 0.931 | 178775356 | 178777049 | 1694 | 58.476 | 52.408 | -0.158 |
| E350 | 1.983311e+02 | 0.014 | 0.974 | 0.998 | 178777149 | 178777317 | 169 | 25.068 | 23.087 | -0.119 |
| E351 | 1.947142e+02 | 0.015 | 0.964 | 0.998 | 178777420 | 178777584 | 165 | 24.701 | 22.994 | -0.103 |
| E352 | 1.890145e+02 | 0.011 | 0.814 | 0.985 | 178777704 | 178777975 | 272 | 24.009 | 22.884 | -0.069 |
| E353 | 1.448864e+02 | 0.003 | 0.837 | 0.987 | 178778874 | 178779118 | 245 | 21.122 | 20.190 | -0.065 |
| E354 | 1.656787e+02 | 0.006 | 0.943 | 0.996 | 178779229 | 178779462 | 234 | 22.670 | 21.427 | -0.081 |
| E355 | 2.075342e+02 | 0.001 | 0.513 | 0.938 | 178780000 | 178780205 | 206 | 24.692 | 24.010 | -0.040 |
| E356 | 1.683999e+02 | 0.009 | 0.914 | 0.994 | 178781121 | 178781263 | 143 | 23.074 | 21.483 | -0.103 |
| E357 | 1.770801e+02 | 0.011 | 0.834 | 0.987 | 178782212 | 178782427 | 216 | 23.785 | 21.886 | -0.120 |
| E358 | 9.834700e+01 | 0.002 | 0.446 | 0.921 | 178782539 | 178782602 | 64 | 17.233 | 16.986 | -0.021 |
| E359 | 1.623808e+02 | 0.002 | 0.511 | 0.938 | 178782806 | 178783064 | 259 | 22.028 | 21.457 | -0.038 |
| E360 | 1.127712e+02 | 0.002 | 0.161 | 0.751 | 178783720 | 178783785 | 66 | 18.091 | 18.340 | 0.020 |
| E361 | 1.682963e+02 | 0.005 | 0.241 | 0.828 | 178784070 | 178784351 | 282 | 22.040 | 22.038 | 0.000 |
| E362 | 1.248969e+02 | 0.007 | 0.326 | 0.879 | 178785620 | 178785742 | 123 | 19.248 | 19.103 | -0.011 |
| E363 | 1.707907e+02 | 0.001 | 0.147 | 0.734 | 178785848 | 178786088 | 241 | 22.142 | 22.146 | 0.000 |
| E364 | 9.823222e+01 | 0.001 | 0.715 | 0.974 | 178786089 | 178786141 | 53 | 17.416 | 16.870 | -0.046 |
| E365 | 2.298763e+02 | 0.002 | 0.558 | 0.947 | 178789360 | 178789497 | 138 | 26.604 | 24.734 | -0.105 |
| E366 | 2.223525e+02 | 0.000 | 0.000 | 0.000 | 178789978 | 178790115 | 138 | 23.543 | 25.816 | 0.133 |
| E367 | 1.764252e+02 | 0.022 | 0.199 | 0.792 | 178790708 | 178790845 | 138 | 21.518 | 23.228 | 0.110 |
| E368 | 3.181350e+02 | 0.006 | 0.976 | 0.998 | 178792072 | 178792197 | 126 | 30.624 | 28.780 | -0.090 |
| E369 | 2.898271e+02 | 0.003 | 0.802 | 0.984 | 178793404 | 178793541 | 138 | 29.408 | 27.559 | -0.094 |
| E370 | 2.246992e+02 | 0.009 | 0.947 | 0.996 | 178794399 | 178794551 | 153 | 26.362 | 24.522 | -0.104 |
| E371 | 1.446189e+02 | 0.009 | 0.757 | 0.978 | 178794922 | 178794954 | 33 | 21.746 | 19.861 | -0.131 |
| E372 | 2.904934e+02 | 0.010 | 0.658 | 0.966 | 178794955 | 178795252 | 298 | 30.098 | 27.263 | -0.143 |
| E373 | 3.524180e+02 | 0.019 | 0.573 | 0.951 | 178798495 | 178799486 | 992 | 30.817 | 30.888 | 0.003 |
| E374 | 4.101340e+02 | 0.007 | 0.770 | 0.980 | 178799487 | 178799731 | 245 | 34.332 | 32.088 | -0.098 |
| E375 | 2.823627e+02 | 0.004 | 0.463 | 0.926 | 178799825 | 178799910 | 86 | 29.492 | 26.983 | -0.128 |
| E376 | 4.408967e+02 | 0.008 | 0.512 | 0.938 | 178800395 | 178800682 | 288 | 35.812 | 32.813 | -0.126 |
| E377 | 2.907381e+02 | 0.004 | 0.667 | 0.967 | 178802138 | 178802286 | 149 | 28.990 | 27.943 | -0.053 |
| E378 | 2.049645e+02 | 0.005 | 0.411 | 0.912 | 178802287 | 178802341 | 55 | 24.447 | 24.066 | -0.023 |
| E379 | 2.287523e+02 | 0.003 | 0.506 | 0.936 | 178804552 | 178804642 | 91 | 25.865 | 25.171 | -0.039 |
| E380 | 1.615059e+02 | 0.001 | 0.613 | 0.959 | 178804643 | 178804655 | 13 | 22.081 | 21.409 | -0.045 |
| E381 | 2.052159e+02 | 0.003 | 0.782 | 0.981 | 178807212 | 178807408 | 197 | 24.871 | 23.807 | -0.063 |
| E382 | 1.425761e+01 | 0.004 | 0.312 | 0.872 | 178807409 | 178807421 | 13 | 6.364 | 6.840 | 0.104 |
| E383 | 1.087470e+00 | 0.065 | 0.235 |  | 178807422 | 178807423 | 2 |  |  |  |
| E384 | 1.123825e+01 | 0.156 | 0.175 | 0.769 | 178817526 | 178817680 | 155 | 4.843 | 6.484 | 0.421 |
| E385 | 1.243821e+01 | 0.191 | 0.062 | 0.559 | 178825313 | 178825495 | 183 | 4.411 | 7.093 | 0.685 |
| E386 | 2.572576e+00 | 0.283 | 0.417 | 0.913 | 178830269 | 178830664 | 396 | 2.295 | 3.142 | 0.453 |
| E387 | 5.886159e+00 | 0.081 | 0.011 | 0.261 | 178830665 | 178830795 | 131 | 2.586 | 5.091 | 0.977 |
| E388 | 1.135931e+00 | 0.043 | 0.155 |  | 178830796 | 178830802 | 7 |  |  |  |
| E389 | 1.352726e+02 | 0.055 | 0.026 | 0.394 | 178833081 | 178834440 | 1360 | 16.438 | 21.679 | 0.399 |
| E390 | 1.422001e+03 | 0.145 | 0.032 | 0.432 | 178835756 | 178836893 | 1138 | 43.238 | 57.810 | 0.419 |
| E391 | 7.396516e+02 | 0.108 | 0.011 | 0.261 | 178836894 | 178837137 | 244 | 32.199 | 46.025 | 0.515 |
| E392 | 1.352747e+03 | 0.130 | 0.021 | 0.358 | 178837138 | 178837744 | 607 | 41.910 | 56.987 | 0.443 |
| E393 | 5.744728e+02 | 0.112 | 0.023 | 0.376 | 178845626 | 178845742 | 117 | 30.066 | 41.355 | 0.460 |
| E394 | 5.116361e+02 | 0.110 | 0.028 | 0.407 | 178850049 | 178850161 | 113 | 28.859 | 39.387 | 0.449 |
| E395 | 5.588848e+02 | 0.118 | 0.031 | 0.424 | 178853441 | 178853624 | 184 | 29.964 | 40.819 | 0.446 |
| E396 | 4.014284e+02 | 0.127 | 0.027 | 0.401 | 178855347 | 178855541 | 195 | 25.428 | 35.809 | 0.494 |
| E397 | 2.488215e+02 | 0.139 | 0.035 | 0.445 | 178856257 | 178856397 | 141 | 20.461 | 29.138 | 0.510 |
| E398 | 2.847899e+02 | 0.120 | 0.027 | 0.402 | 178865767 | 178865916 | 150 | 21.838 | 30.896 | 0.501 |
| E399 | 4.124365e+02 | 0.144 | 0.027 | 0.402 | 178868026 | 178868205 | 180 | 25.257 | 36.373 | 0.526 |
| E400 | 4.566186e+02 | 0.136 | 0.033 | 0.435 | 178869117 | 178869305 | 189 | 26.911 | 37.782 | 0.489 |
| E401 | 3.581690e+02 | 0.158 | 0.031 | 0.427 | 178871427 | 178871552 | 126 | 23.638 | 34.302 | 0.537 |
| E402 | 4.079773e+02 | 0.165 | 0.027 | 0.399 | 178872133 | 178872312 | 180 | 24.726 | 36.340 | 0.556 |
| E403 | 4.535625e+01 | 0.021 | 0.014 | 0.292 | 178873021 | 178877963 | 4943 | 10.061 | 12.640 | 0.329 |
| E404 | 2.666923e+02 | 0.167 | 0.041 | 0.478 | 178877964 | 178878143 | 180 | 21.028 | 30.077 | 0.516 |
| E405 | 1.787827e+02 | 0.179 | 0.050 | 0.515 | 178884901 | 178884951 | 51 | 17.486 | 25.134 | 0.523 |
| E406 | 2.810616e+02 | 0.186 | 0.044 | 0.491 | 178884952 | 178885092 | 141 | 21.387 | 30.829 | 0.528 |
| E407 | 2.733323e+02 | 0.186 | 0.045 | 0.497 | 178886752 | 178886871 | 120 | 21.205 | 30.425 | 0.521 |
| E408 | 2.944426e+02 | 0.153 | 0.028 | 0.405 | 178888527 | 178888668 | 142 | 21.626 | 31.535 | 0.544 |
| E409 | 2.952483e+02 | 0.141 | 0.016 | 0.317 | 178905329 | 178905501 | 173 | 21.047 | 31.767 | 0.594 |
| E410 | 1.342997e+00 | 0.469 | 0.853 |  | 178915751 | 178915807 | 57 |  |  |  |
| E411 | 3.147764e+02 | 0.161 | 0.017 | 0.323 | 178918713 | 178918907 | 195 | 21.248 | 32.787 | 0.626 |
| E412 | 0.000000e+00 |  |  |  | 178926512 | 178926665 | 154 |  |  |  |
| E413 | 2.105547e+02 | 0.150 | 0.035 | 0.447 | 178944535 | 178944651 | 117 | 18.775 | 27.101 | 0.530 |
| E414 | 2.618217e+02 | 0.130 | 0.031 | 0.423 | 178961230 | 178961483 | 254 | 20.966 | 29.796 | 0.507 |
| E415 | 1.879457e+02 | 0.160 | 0.040 | 0.473 | 178975057 | 178975165 | 109 | 17.807 | 25.748 | 0.532 |
| E416 | 3.475175e+02 | 0.167 | 0.043 | 0.486 | 178978484 | 178978675 | 192 | 23.882 | 33.678 | 0.496 |
| E417 | 3.198790e+02 | 0.151 | 0.034 | 0.442 | 179047284 | 179047406 | 123 | 22.806 | 32.569 | 0.514 |
| E418 | 2.627019e+02 | 0.127 | 0.029 | 0.416 | 179049840 | 179050059 | 220 | 21.007 | 29.836 | 0.506 |
| E419 | 1.340349e+00 | 0.078 | 0.614 |  | 179050060 | 179050063 | 4 |  |  |  |
| E420 | 6.665750e-01 | 0.138 | 0.726 |  | 179050064 | 179050086 | 23 |  |  |  |

Gene: ***TTN*** ENSG00000155657. Chromosome 2: 178,525,989-178,830,802 reverse strand.

Gene: ***CCDC141*** ENSG00000163492. Chromosome 2: 178,829,757-179,050,086 reverse strand.

**Online References**

1. Vikhorev PG, Smoktunowicz N, Munster AB, Copeland O, Kostin S, Montgiraud C, Messer AE, Toliat MR, Li A, dos Remedios CG, Lal S, Blair CA, Campbell KS, Guglin M, Richter M, Knoll R, Marston SB. Abnormal contractility in human heart myofibrils from patients with dilated cardiomyopathy due to mutations in TTN and contractile protein genes. *Scientific Reports* 2017;**7**:14829.

2. Vikhorev PG, Ferenczi MA, Marston SB. Instrumentation to study myofibril mechanics from static to artificial simulations of cardiac cycle. *MethodsX* 2016;**3**:156-170.

3. Witayavanitkul N, Ait Mou Y, Kuster DW, Khairallah RJ, Sarkey J, Govindan S, Chen X, Ge Y, Rajan S, Wieczorek DF, Irving T, Westfall MV, de Tombe PP, Sadayappan S. Myocardial infarction-induced N-terminal fragment of cardiac myosin-binding protein C (cMyBP-C) impairs myofilament function in human myocardium. *The Journal of Biological Chemistry* 2014;**289**:8818-8827.

4. de Tombe PP, Stienen GJ. Protein kinase A does not alter economy of force maintenance in skinned rat cardiac trabeculae. *Circulation Research* 1995;**76**:734-741.

5. de Tombe PP, Stienen GJ. Impact of temperature on cross-bridge cycling kinetics in rat myocardium. *The Journal of Physiology* 2007;**584**:591-600.

6. Vikhorev PG, Vikhoreva NN. Cardiomyopathies and related changes in contractility of human heart muscle. *International Journal of Molecular Sciences* 2018;**19**:2234.

7. McNamara JW, Li A, dos Remedios CG, Cooke R. The role of super-relaxed myosin in skeletal and cardiac muscle. *Biophysical Reviews* 2015;**7**:5-14.
